# *In-Vivo* Real-Time Control of Protein Expression from Endogenous and Synthetic Gene Networks

**DOI:** 10.1371/journal.pcbi.1003625

**Published:** 2014-05-15

**Authors:** Filippo Menolascina, Gianfranco Fiore, Emanuele Orabona, Luca De Stefano, Mike Ferry, Jeff Hasty, Mario di Bernardo, Diego di Bernardo

**Affiliations:** 1 TeleThon Institute of Genetics and Medicine (TIGEM), Naples, Italy; 2 Department of Electrical Engineering and Information Technology, University of Naples Federico II, Naples, Italy; 3 Institute of Microelectronics and Microsystems (IMM), CNR, Naples, Italy; 4 Department of Bioengineering, University of California, San Diego, La Jolla, California, United States of America; 5 Department of Engineering Mathematics, University of Bristol, Bristol, United Kingdom; Johns Hopkins University, United States of America

## Abstract

We describe an innovative experimental and computational approach to control the expression of a protein in a population of yeast cells. We designed a simple control algorithm to automatically regulate the administration of inducer molecules to the cells by comparing the actual protein expression level in the cell population with the desired expression level. We then built an automated platform based on a microfluidic device, a time-lapse microscopy apparatus, and a set of motorized syringes, all controlled by a computer. We tested the platform to force yeast cells to express a desired fixed, or time-varying, amount of a reporter protein over thousands of minutes. The computer automatically switched the type of sugar administered to the cells, its concentration and its duration, according to the control algorithm. Our approach can be used to control expression of any protein, fused to a fluorescent reporter, provided that an external molecule known to (indirectly) affect its promoter activity is available.

## Author Summary

A crucial feature of biological systems is their ability to maintain homeostasis in spite of ever-changing conditions. In engineering, this ability can be embedded in devices ranging from the thermostat to the autopilot of a modern plane using control systems which operate via a negative feedback mechanism: the quantity to be controlled is measured then subtracted from the desired reference value, and the resulting error is used to compute the control action to be implemented on the physical system (e.g. switching on or off the heating, changing the position of the rudder). Here, we developed and applied a method to regulate the expression level of a protein, in a growing population of cells over several generations, in a completely automatic fashion. We designed and implemented an integrated platform comprising a microfluidic device, a time-lapse microscopy apparatus, and a set of motorized syringes, all controlled by a computer. We tested the platform to force yeast cells to express a desired time-varying amount of a gene in yeast. Our method can be applied to control a protein of interest in vivo allowing to probe the function of biological systems in unprecedented ways.

## Introduction

**Figure 1:**
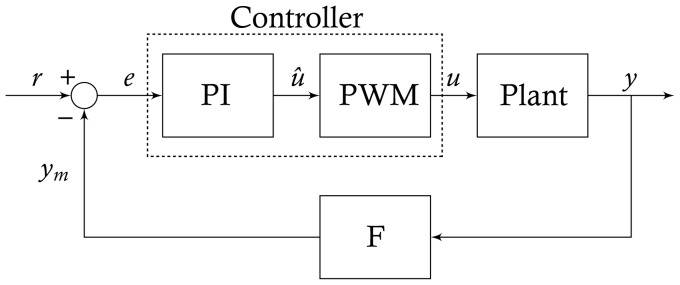
Feedback control block scheme. The controller consists of a Proportional-Integral (PI) block followed by a Pulse Width Modulation (PWM) block encoding of the control input 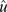. The PWM transforms the continuous control action 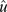 into a train of rectangular pulses 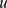, which represents either Galactose (high) or Glucose (low). The alternating series of glucose and galactose pulses is applied to the cell population to be controlled (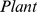), whose output 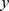 (the controlled variable) is filtered (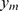) by a low-pass filter (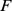) before being fed back to the controller. The difference between 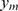 and its desired reference level 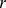, namely the error 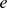, is used by the PI controller to compute the control input to be supplied to the system to minimize the error signal 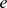.

A crucial feature of biological systems is their ability to maintain homeostasis in spite of ever-changing environmental and intracellular conditions. In man-made systems, this ability can be engineered in devices ranging from the simple thermostat to the complex autopilot of a modern plane using “controllers”, which operate via a simple “negative feedback” mechanism (Figure 1): the quantity to be controlled (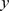) is measured (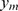) via a sensor (whose dynamics are described by 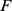), then subtracted from the desired reference value (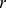) (i.e. negative feedback), and the resulting error (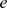) is used by the controller to compute the “control action” (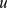) to be implemented (or actuated) on the physical system (e.g. switching on or off the heating, changing the angular position of the rudder).

Control engineering has been applied as a powerful theoretical framework to elucidate the underlying principles driving gene networks [1]–[4], to predict their dynamics and their robustness to noise [5], [6], and to theoretically demonstrate the possibility of steering gene network dynamics [7]–[9].

More recently, other groups have reported experimental applications of control engineering to drive gene expression from artificial inducible promoters by means of external stimuli (e.g. light or osmotic pressure) either in single cells, or across a cell population [10]–[12]. Toettcher and colleagues documented a successful application of closed-loop optogenetic control of membrane recruitment system in mammalian cells (characteristic time of process in the order of seconds) [11]. Milias-Argeitis and colleagues showed how population level control of a light-switchable gene system (characteristic time in the order of minutes) can be achieved by manually sampling a liquid culture of *S. cerevisiae*, then estimating target protein concentration via flow cytometry and control this quantity via Model Predictive Control and Kalman Filtering [12]. While this manuscript was under review, Uhlendorf and colleagues reported a successful attempt to drive gene expression from the Hog1 promoter in yeast using as control input changes in osmolarity [13]. These authors coupled an external feedback strategy to microscopy and microfluidics to achieve fully automated external control of the reporter fluorescent protein up to 15 hrs.

Here we present, for the first time, the development and application of an automatic control system to regulate at will the level of expression of a protein from the *GAL1* endogenous promoter in an exponentially growing population of yeast cells. We also demonstrated the ability of the control system to regulate at will protein expression from a complex synthetic transcriptional network. We controlled protein expression level by changing in real-time the concentration of a set of inducer molecules, known to modulate its expression (i.e. galactose and glucose).

**Figure 2:**
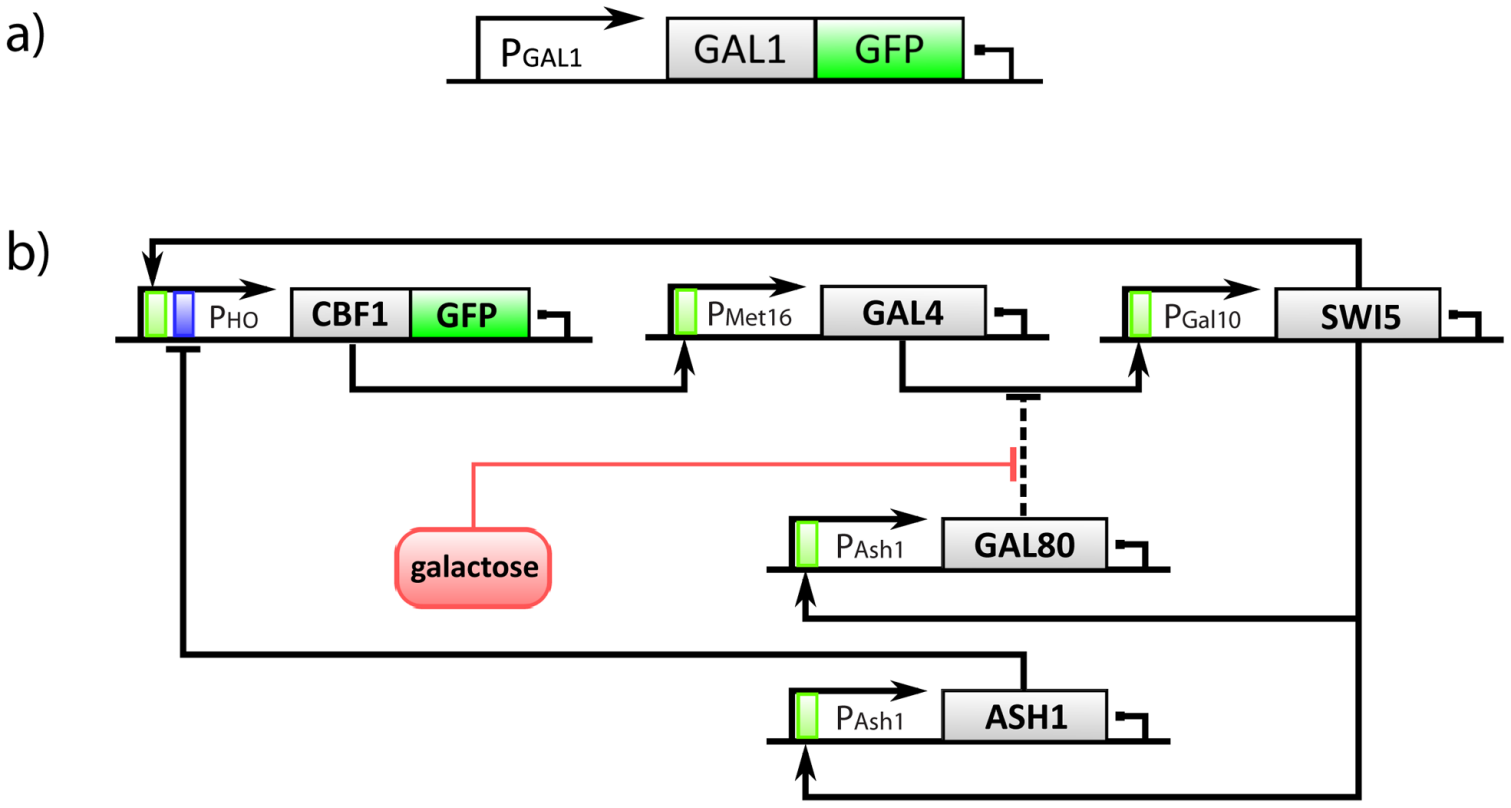
Biological systems. (a): The Gfp protein was integrated downstream of the endogenous *GAL1* promoter (yeast strain courtesy of Prof. Botstein lab). (b): IRMA is composed of 5 genes encoding for transcription factors modulating the expression of each other. Both the transcription factors in the network and the promoters driving their expression are shown (adapted from [17]). Solid lines model transcriptional interactions, while dashed lines are meant to represent protein-protein interactions.

We first demonstrated the ability of our control platform to regulate the level of expression of a reporter protein fused to the Gal1p protein from the endogenous *GAL1* promoter (Figure 2a). We then applied the same control platform to regulate the level of expression in a complex synthetic gene network (Figure 2b), where the inducer molecule (galactose) directly activates the transcription factor, Gal4p, which in turns drives the expression of another transcription factor (Swi5p), which finally binds the promoter driving the expression of the reporter protein (Cbf1p-Gfp). Due to these multiple transcriptional/translational steps (Gal4p 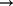 Swi5p 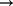 Cbf1-Gfp), the system is much slower compared to simple promoter-reporter systems, thus control becomes much more challenging due to the delay introduced by this indirect regulation.

Our approach is applicable to a large class of gene networks to control expression of a protein of interest from an endogenous promoter, provided that: (*a*) an external molecule known to affect (even indirectly) the promoter activity is available; (*b*) a fluorescent reporter is fused to the protein; and (*c*) either a mathematical model of the gene network is available or dynamical properties of the bioprocess are compatible with a PI-PWM control configuration (see “Control Objective and Control Strategy” and Figures S7–S8).

## Materials and Methods

### Microfluidic master mold and devices fabrication

The microfluidic device presented in [14] has been used to trap cells and perform experiments. To this aim, a master mold has been produced using a 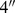 (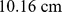) silicon wafer as substrate (Silicon Valley Microelectronics, US). In order to develop this device, we used multilayer soft-lithography with SU-8 (Microchem, US) as photoresist. Once the mold was ready we used (Tridecafluoro-1,1,2,2-Tetrahydrooctyl)-1-Trichlorosilane (Sigma-Aldrich, US) to prevent polymer from sticking to microstructures; at this point replica molding allowed us to obtain functional devices (see Supplementary Information).

### Experimental setup

The experimental setup is the same for both strains of cells used in this study (yGIL337 and IC18). Batch cultures were cultured for 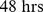 in Synthetic Complete + Galactose (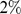) + Raffinose (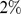) and repeatedly diluted. On the day designated for the actual control experiment, syringes featuring both Synthetic Complete + Glucose (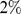) and Synthetic Complete + Galactose (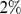) + Raffinose (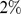) were connected to the device; syringes filled with ddH2O were attached to sink ports. Media and sugars filled syringes were attached to a computer controlled linear guide; the initial position for the syringes was 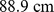 above the level of the device, while the ddH2O syringes were set at 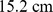. Hydrostatic pressure drove the flow of media in the device. We then loaded cells into the microfluidic device. Visual inspection at 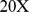 and 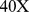 magnifications allowed to exclude the presence of air bubles in the channels. At this point, the imaging field was set on the cell trap (see [14] for references) and the control algorithm was started. In depth details concerning this procedure are reported in Supplementary Information.

### Microscopy and image processing

Microscopy image acquisition has been carried out by with NIS Elements v. 3.22 software. Phase contrast and fluorescent images were acquired at intervals of 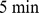. The control algorithm was synchronized with the acquisition process by running a polling routine that checked for the presence of new files in a predefined folder. Once a new set of images was found, the control algorithm run an image processing sub-routine meant to segment the phase contrast image (see Supplementary Information for more details), locate the cells and obtain a binary mask that was used to select only pixels belonging to cells in the field. This mask was employed in the calculation of the population average fluorescence as reported in Supplementary Information. Our control scheme then used this value as a readout of the 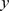 signal and then proceeded to the computation of the input action 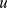.

## Results

### Choice of biological systems to control

#### Gal1 promoter in *S. cerevisiae*

In order to test our control platform on an endogenous promoter in *S. cerevisiae*, we selected the *GAL1* promoter driving expression of the Gal1p protein. In order to follow in real-time the expression level of Gal1p, we used a strain of yeast cells (yGIL337, Gal1-GFP::KanMX, Gal10-mCherry::NatMX) constructed by Lang et al. [15] in which the Gal1 protein, expressed by the *GAL1* promoter, was fused to a green fluorescent protein (Gfp), as shown in [15] (Figure 2a).

The actvity of the *GAL1* promoter is governed by the presence of galactose in the cells' growing medium. This sugar is interpreted as a “switch on” signal for the expression of the *GAL1* gene; when yeasts are fed with glucose the production of Gal1 protein is repressed [16]. Yeast cells will first consume all the available glucose in the medium and then switch to galactose. Hence, the control input can either be glucose (switch off signal) or galactose (switch on signal), but not an intermediate concentration of the two, because cells will not respond to galactose when glucose is present.

#### IRMA synthetic network in *S. cerevisiae*

IRMA (In-vivo Reverse engineering Method Assessment) is a synthetic network we previously constructed in yeast *S. cerevisiae* and shown in Figure 2 b [17]. It consists of 5 genes regulating each other via positive and negative feedback loops, and represents one of the most complex synthetic networks built so far [18]. The Cbf1-Gfp fusion protein is expressed from the *HO* promoter controlled by two transcription factors: a cell cycle-independent Swi5p mutant (swi5AAA) and Ash1p. The network comprises a transcriptional positive feedback loop from *CBF1* back to itself, via *GAL4* and *SWI5*; and a transcriptional negative feedback loop via *ASH1*. A further regulation is present between *GAL80*, *GAL4* and *SWI5*, whose expression is driven by the *GAL10* promoter, bound by GAL4p. The network can be “switched on” by administering Galactose (GAL) in the medium, which allows *SWI5* to be transcribed by the *GAL10* promoter, or “switched off” by Glucose.

Of note, *CBF1-GFP* expression is delayed with respect to the other genes [17]. This delay is due to the sequential recruitment of chromatin-modifying complexes at the *HO* promoter, which follow binding of Swi5p and other transcription factors [19], and it is estimated in the range of 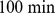 [17].

Galactose and Glucose can be used to control the network's dynamics, which, in turn, can be tracked by estimating the fluorescence level of Cbf1-Gfp, one of IRMA's proteins. By taking full advantage of the detailed description of the biomolecular processes provided in [17], [19] we derived a Delay Differential Equations based model (described in Supplementary Information) that was able to capture, to a reasonable extent, the dynamics of the gene network [17].

Interestingly, IRMA dynamical properties are commonly observed in endogenous gene regulatory networks and pathways. IRMA contains two of the most common regulatory motifs found in eukaryotic cells, i.e. positive and negative transcriptional feedbacks loops [20]. Moreover, a protein-protein regulatory interaction is also present, which is much faster than transcriptional regulatory interactions, thus adding concurrent dynamics at different time-scales typical of endogenous regulatory networks. These properties pose two main theoretical and practical challenges: (a) devising a control algorithm for hybrid dynamical system switching between two highly non-linear/time-delayed subsystems; (b) developing a technolgical platform allowing to probe and control the transcriptional/translational activity of a cell. IRMA is therefore an ideal benchmark for testing our in-vivo control strategy on a complex gene network [17], [21].

### Control objective and control strategy

#### GAL1 promoter

The control objective consists in driving a growing population of yeast cells to produce a desired amount of the Gal1-Gfp fusion protein, over thousands of minutes, by automatically switching the type of sugar administered to the cell, its concentration and its duration.

**Figure 3:**
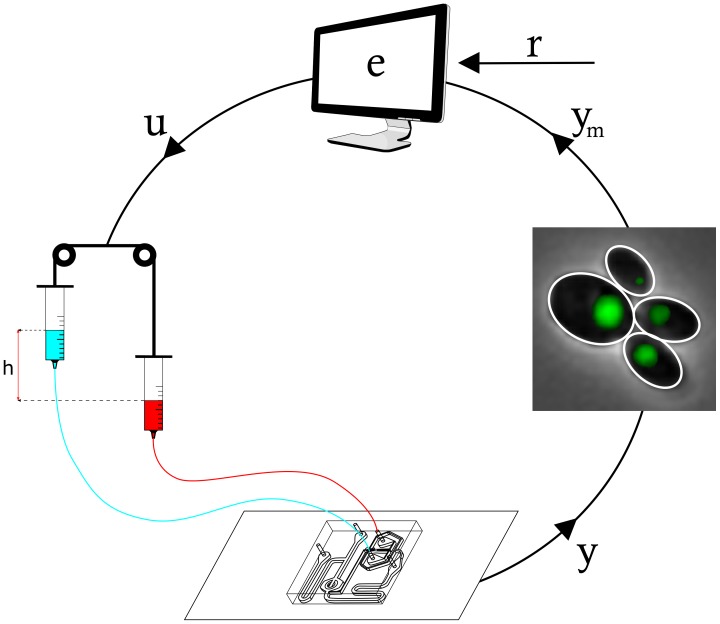
Technological platform enabling in-vivo control experiments. In-vivo control experiment were carried out via a microfludics-based approach, featuring a computer implementation of the control algorithm and an inverted microscope to sense the fluorescence signal. The computer uses the images taken by the automated microscopy unit to quantify cells fluorescence (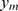) and compare it with the desired amount 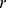. Once the control algorithm has computed the control action 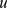 on the basis of the result of the previous comparison (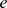), it varies the height of two syringes filled with either Galactose or Glucose. Hydrostatic pressure generated by the relative difference in the heights of the two syringes drives the flow in the microfluidic device and determines the type of sugar cells will sense within the chamber. Images are sampled at intervals of 5 minutes and used by the control algorithm to close the control loop.

In order to achieve the control objective, we designed a simple control law to automatically regulate the administration of Galactose/Glucose to the cells (input 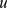) as a function of the amount of fluorescent protein produced by the yeast population (output 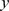), following the scheme in Figure 3.

The input acts nonlinearly on the dynamics of the network, since, as soon as the cells sense Glucose in the medium, they stop responding to Galactose. Therefore, the control input is restricted to be binary, i.e. either Galactose (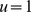) or Glucose (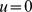). The system output 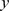 is the level of the green fluorescent protein fused to *Gal1p* (Gal1-Gfp), which can be used as a proxy of the protein concentration [22].

Given the uncertain nature of biological systems and the difficulty, or impossibility, in deriving precise quantitative mathematical models, we decided to design a simple yet effective classic control scheme, which does not require detailed knowledge of the system to be controlled.

Proportional-Integral (PI) controllers use the error 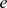 in Figure 1 to compute the control action, which is proportional to the present error (P) and to the past evolution of the error (I) (Supplementary Information). PI controllers are widely used in industry, and have been shown to be effective also in controlling protein localization and signal transduction activity in single cells [11].

Since the control input 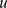 is binary, a further step is needed to convert the output of the PI block (which is proportional to the error and its integral) to a binary signal. It is impossible, at this stage, to avoid the analogy with the problems faced in the design of feedback control strategies for power electronic circuits [23]. Here, switches and SCRs (silicon controlled rectifiers) can only be turned on or off, some output is typically measured or estimated and, particularly in industrial applications, compensating noise and external disturbances is of utmost importance. The simplest and most widely used technique in this context is PWM (Pulse Width Modulation) control; this is also the encoding strategy we implemented in the control scheme to control the gene network in the yeast population (Figure 1 and Figure S2).

The idea behind PWM is to encode a continuous time-varying signal as a train of rectangular pulses, whose duration is proportional to the amplitude of the encoded signal. In its simplest implementation, which we used here, a periodic sawtooth wave (with period equal to 10 min) is compared with the output of the PI block in order to modulate the width of each pulse [24].

We used a simple mathematical description of the GAL1 promoter to tune the gains of the PI regulator and the parameters of the PWM module (Supplementary Information).

One of the most difficult aspects of controlling a biological system, which lies at the core of our work, is to devise an experimental platform enabling such a strategy to be applied to living cells, as described in the next section.

#### IRMA network

The control objective is the same as for the Gal1 promoter previously described, i.e. controlling the level of expression of the reporter protein (Cbf1-Gfp). However, in the IRMA network, unlike the GAL1 promoter system, the *CBF1-GFP* gene is not under the direct control of the inducer molecule (i.e. glucose or galactose). Indeed, as shown in Figure 2b, galactose activates Gal4p, which then drives the expression of *Swi5p* that ultimately binds the *Gal10* promoter driving Cbf1p-GFP expression. This adds a considerable delay in the Cbf1-Gfp activation following galactose treatment [17].

IRMA can be described as a single input-single output nonlinear time-delayed dynamical system, where the input 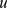 models the presence/absence of Galactose and the output 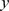 is the concentration of one of its proteins, namely Cbf1-GFP.

The control algorithm designed for this system, as we did for the *Gal1* promoter, is based on a PI regulator whose output is encoded in a switching signal via a PWM module (see Supplementary Informations for details).

However, in order to compensate for the estimated delay of 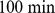 in the Gal1-Gfp gene transcription, which may introduce unwanted oscillations in the controlled variable (i.e. Gfp fluorescence level), we designed and implemented a version of the controller including a predictor inspired by the classic scheme proposed by Otto Smith [25].

In particular, IRMA's mathematical model [17] was used to predict, via simulation, the network behavior by removing the delay from the model equation. This simulated response is then used in real-time by the controller to compute the error (Supplementary Information).

Numerical simulations show that this predictor block, however, is not strictly necessary for control; specifically, when the delay is short compared to the desired dynamics of the reference signal, the predictor can be completely removed from the control scheme (Supplementary Information and Figures S7–S8).

**Figure 4:**
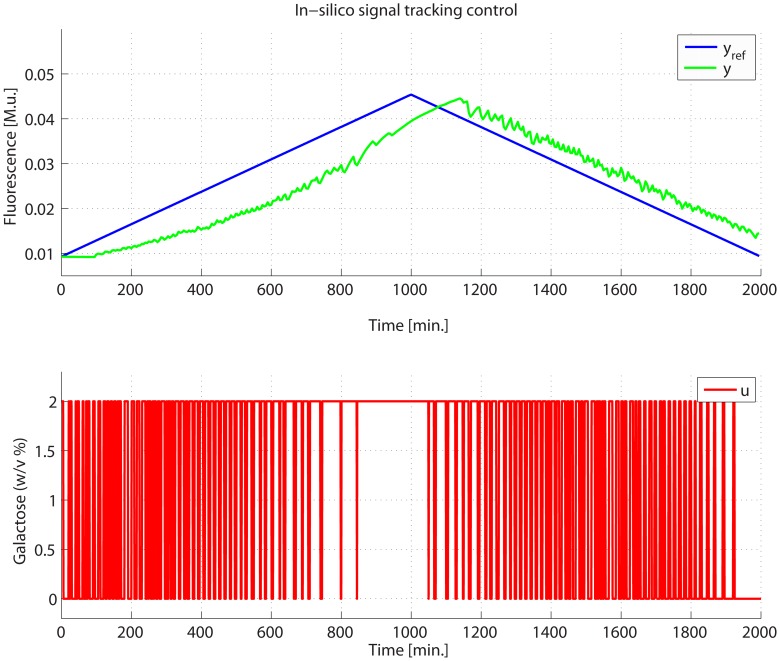
In-silico control of IRMA. The PI-PWM-Predictor control scheme has been tested in-silico, using the non-linear IRMA model as a proxy, in order to evaluate its performances before implementing it in in-vivo experiments. In this experiment, the CBF1-GFP protein (green line) was controlled so as to follow a triangular reference signal (blue line). The optimal input signal 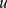 computed by the control algorithm is reported in red: it is a square wave encoding Galactose (higher state) and Glucose (lower state). The input binary variable has been rescaled in this plot for the sake of readability.

 The PI-PWM control law (with and without the predictor) was designed and simulated in-silico, in order to evaluate its performance before implementation, and to empirically select the control parameters. Two different experiments were used to assess the performance of the controller: (1) *set-point regulation*, where cells are forced to produce a fixed desired amount of Cbf1-Gfp protein; (2) *signal tracking*, where yeasts are required to synthesize a target time-varying amount of the controlled protein. *In-silico* results of both types of experiments are presented in Figure 4, Figures S5–S6–S7–S8 and in Supplementary Information.

*In-silico* results show that both set point and tracking experiments can in principle be successfully carried out by the selected control strategy.

### Implementation of the PI-PWM controller for in-vivo control of protein expression

For the in-vivo control implementation, we designed and implemented an integrated platform based on a microfluidic device, a time-lapse microscopy apparatus, and a set of actuated syringes, all controlled by a computer, as depicted in Figure 3.

At the core of this platform lies a microfluidic chip [14]. The chip has a micro-chamber (height: 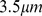) which “traps” yeasts, which can only grow in a monolayer, thus making their automated image analysis easier. Once loaded in the device, yeasts can be exposed to any combination of two inducer compounds by simply modulating the difference in hydrostatic pressures at the two inlets, thanks to the Dial-a-Wave system [14] (Supplementary Information). This can be easily achieved by varying the vertical position of syringes filled with sugar-supplemented media using motorized linear rails. A Finite State Automaton (FSA), implementing the control logic, runs on a computer and, at intervals of 5 minutes, analyses the images automatically captured by the microscope hosting the microfluidic chip. A custom image processing algorithm locates the yeast cells in phase contrast and quantifies the population average Gfp intensity (Supplementary Information and Figure S9). This information is then used by the controller to compute the relative duration of Galactose/Glucose pulses, which must be applied to meet the control objective.

### Experimental results

#### GAL1 promoter

The control experiment consisted in a set-point control task, i.e. forcing yeast cells to reach and maintain a constant level of fluorescence equal to 50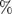 of their maximum fluorescence level when grown in galactose-rich medium. Specifically, after having grown yeast cells overnight in galactose, cells were placed in the microfludics device, part of the control platform, for a calibration phase of 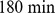 during which cells were kept in galactose to estimate the average maximum Gfp fluorescence level expressed by the cell population. The desired value was then set to 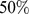 of the estimated maximum fluorescence value.

**Figure 5:**
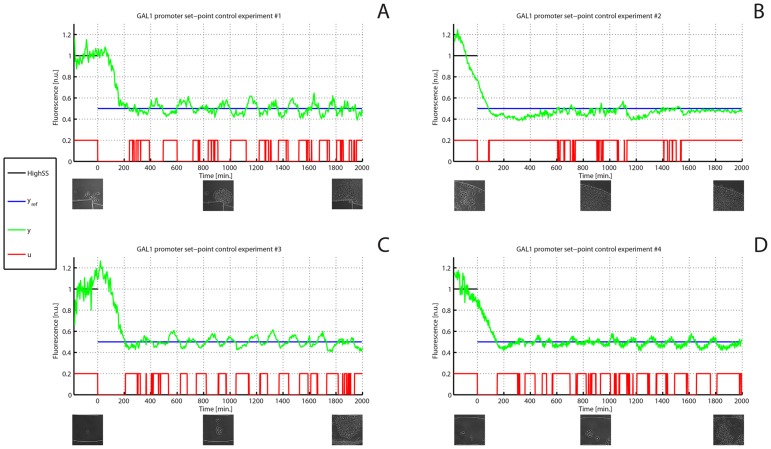
*In-vivo* set point control experiments on the GAL1 promoter. (A–D) Four *in-vivo* set point control experiments were performed on the GAL1 promoter. The desired (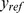 in blue) and experimentally quantified GFP fluorescence (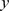 in green) in the cell population are shown for the whole duration of the experiments; the control action starts at time 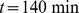 and lasts for 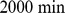. The fluctuations in fluorescence during the 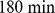 calibration phase are due to stress response after loading cells in the microfludics device. The input signal 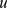, computed in real-time by the control algorithm, is shown in red: a high signal corresponds to galactose-rich growth medium, a low signal to glucose growth medium. (Insets) Images taken during the experiments show the growing yeast populations at the beginning, at the half and at the end of each experiment.

As shown in Figure 5 the control action works effectively in keeping the output, namely the measured fluorescence, close to the desired set-point for 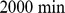. Despite the increasing number of cells and the cell-to-cell variability intrinsic to gene expression, the control error remained bounded for the whole experiment (Figure S10, S11, S12, S13 and S14 of Supplementary Information).

To assess the effectiveness of the feedback control strategy we performed two different types of “negative control” experiments: (1) an additional set-point control experiment, as described before, but with the difference that as soon as the Gfp reached the desired set-point value (i.e. 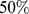 of the maximum fluorescence) the control algorithm was stopped and the input was switched randomly between galactose and glucose (Supplementary Information); (2) yeast cells were fed for 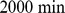 only with galactose (sustained “ON” input).

**Figure 6:**
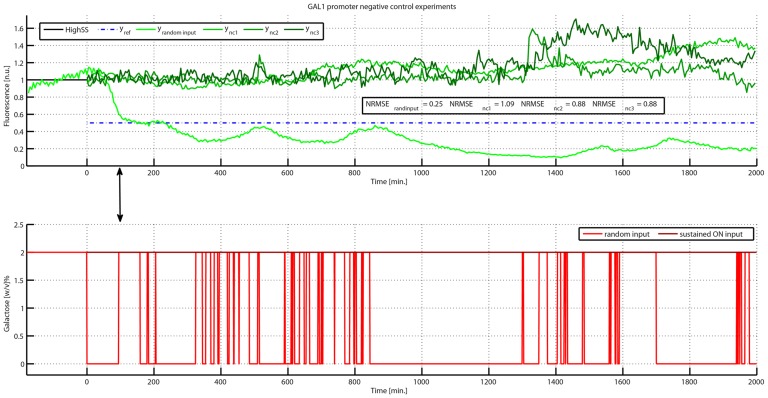
*In-vivo* negative control experiments on the GAL1 promoter. (Top panel) the three green signals (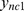, 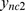 and 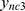) represent the measured GFP fluorescence in the cell population for a constant concentration of galactose. The desired (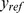 in blue) and experimentally quantified GFP fluorescence (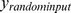 in light green) for the whole duration of the random input negative control experiments are also shown. For this experiments, the control action starts at time 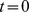 and it is stopped at time 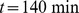 as soon as the GFP reaches the desired set-point (arrow), from this time instant onwards, the input randomly switched between galactose and glucose, as described in the Supplementary Information. (Bottom panel) the dark red line represents the constant concentration of galactose (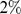) provided to cells corresponding to the experiments 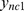, 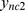 and 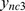; the light red series of pulses, corresponding to the experiment 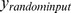, represents the closed loop control input calculated by the control algorithm starting from time 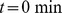 up to time 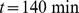, as indicated by arrow; from this time onwards, the input randomly switched between glucose and galactose (Supplementary Information).

The results of the negative control experiments are shown in Figure 6. It can be appreciated that, as expected, when cells were kept in constant galactose (Figure 6) the measured GFP fluctuated and diverged from the initial value; whereas, when a random input was applied (Figure 6) the output of the system diverged from the desired value, responding to the series of galactose/glucose pulses provided to cells.

These experiments convincingly demonstrate the ability of our control platform to achieve and maintain a desired level of fluorescence by steering gene expression dynamics across the yeast cell population.

#### IRMA network

**Figure 7:**
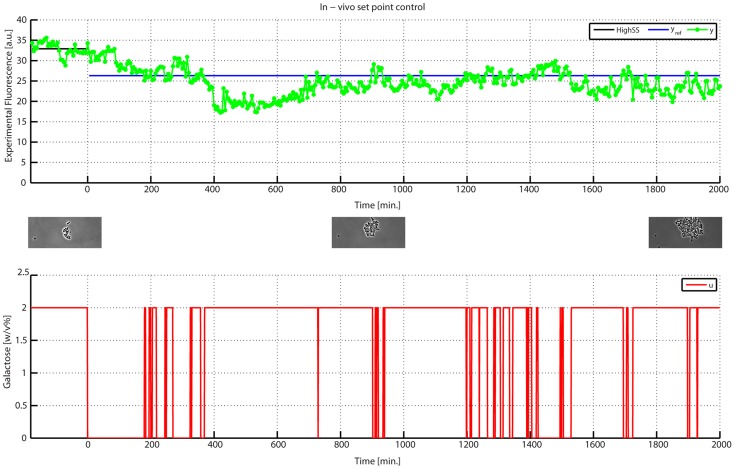
*In-vivo* set point control experiment on IRMA. (Top panel) the desired (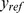 in blue) and experimentally quantified GFP (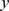 in green) are shown for the whole duration of the experiment; the control action starts at time 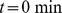 and lasts for 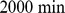. The p-value estimates the statistical significance of the control action in maintaining the desired set-point (Supplementary Material). (Bottom panel) the input signal 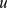 computed by the control algorithm is shown in red. (Insets) Images taken during the experiment show the growing yeast population at the beginning, at the half and at the end of the experiment.

In order to test the control scheme in a more complex setting, we performed a set-point control experiment in the IRMA network, where the cell population was required to reach and maintain a fluorescence level equal to 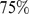 of its maximum value in Galactose over a time interval of 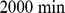 (Figure 7, in Figure S15 and Video S2).

In this experiment, we adopted a simple PI-PWM control, as in the case of the Gal1 promoter (Supplementary Information). The experiment started with a short calibration phase of 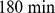 in Galactose to estimate the maximum Cbf1-Gfp fluorescence level produced by the cell population.

As shown in Figure 7 and in Figure S15 the desired fluorescence level was successfully achieved and maintained for over 24 hours, the control error did not diverge and remained bounded around zero. The cell-to-cell variability, estimated using the CV, did not change appreciably throughout the experiment, and was found in the expected range [26], despite the increase in the number of cells (estimated from 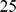 to 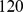 cells; Figure S16 and Video S2). As expected, however, due to the more complex network, the fluctuations around the set-point are more evident. Indeed the Normalised Root Mean Squared Error (NRMSE), reported in Figure S15 is greater in this case when compared to the Gal1 promoter control experiment in Figure S10.

 For comparison in Figure S17 and S18, we also reported two experiment without control input, showing that without active control protein expression fluctuates during the course of the experiment.

**Figure 8:**
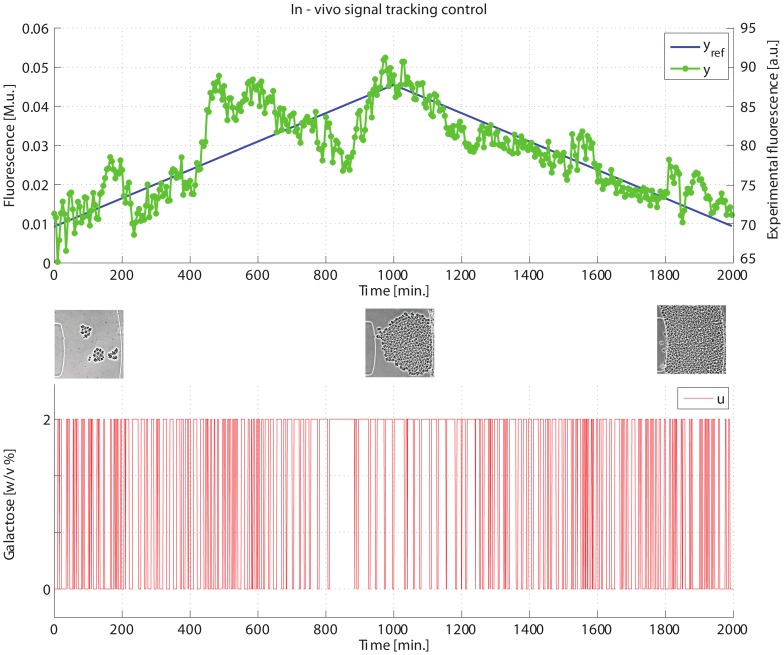
*In-vivo* signal tracking control experiment on IRMA. (Top panel) the desired (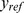 in blue) and experimentally quantified GFP (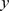 in green) are shown for the whole duration of the experiment (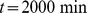). (Bottom panel) the input signal 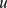 computed by the control algorithm is shown in red. (Insets) Images taken during the experiment show the growing yeast population at the beginning, at the half and at the end of the experiment.

In order to test the PI-PWM-predictor control scheme, we set up a more challenging signal tracking task, where the Cbf1-Gfp level of the cell population was required to track a triangular wave over a time interval of 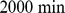 corresponding to more than 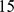 generations of a yeast cell (approx. cell cycle of 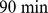), as shown in Figure 8 (blue line). At the peak of the triangular wave, cells were required to express a fluorescence level equal to 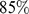 of the maximum level they could produce.

To estimate the minimum and maximum fluorescence level, which can vary according to the experimental conditions, the control experiment started with an initial calibration phase, where the network was switched off by administering Galactose (Supplementary Information and Figures S24–S25).

The calibration phase was needed to establish the minimal (in Glucose) and maximal (in Galactose) Cbf1-Gfp fluorescence level produced by the cell population. This range was then used by the control algorithm to calibrate the predictor block and to set the desired level of fluorescence. The calibration phase lasted 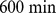, (Galactose for 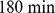 and Glucose for 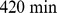). (Supplementary Information and Figures S24–S25).

As shown in Figure 8, Figure S19 and Video S1, the proposed strategy effectively accomplishes the goal of controlling protein expression in a complex gene network across a population of yeast cells, by means of automatic administration of inducer molecules.

Remarkably, the designed control scheme proves to be sufficiently robust despite the large variation in the number of cells over the experiment duration (estimated from 100 to 1200 cells; Figure S20) and despite the inevitable biological noise. Biological noise is a well-known phenomenon that causes genetically identical cells to respond differently to the same input [26]. We quantified biological noise by estimating the standard deviation and the Coefficient of Variation (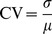) throughout the experiment (Figures S19–S20); despite the number of cells increasing of an order of magnitude during the experiment (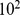 to 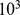) the CV does not change considerably, and its level is well in the expected range for living cells [26]. Hence the population of cells is entrained by the control signal which keeps them from deviating from the reference signal, as can be appreciated by observing the time evolution of the control error (difference between the average fluorescence value of the population and the desired reference value) in Figure S21.

Of note, the results are even better than what expected from simulations (Figure 4), in terms of the offset between the reference signal and the actual fluorescence level. The improvement in the experiments can be explained by considering the delay term present in the mathematical model of IRMA used in the simulations. Such delay models the time required for the activation of the *HO* promoter driving expression of the *CBF1* gene in the network, that was quantified to be equal to 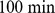 [17]. The improvement in the experiments is explained by observing that real cells exhibit a much smaller delay than expected.

A possible explanation of this effect can be found in the epigenetic control of the *HO* promoter; the PI controller keeps activating this promoter indirectly via quickly alternating Galactose and Glucose, thus preventing the promoter to be completely silenced via chromatin remodeling, thus considerably reducing the transcriptional delay.

**Figure 9:**
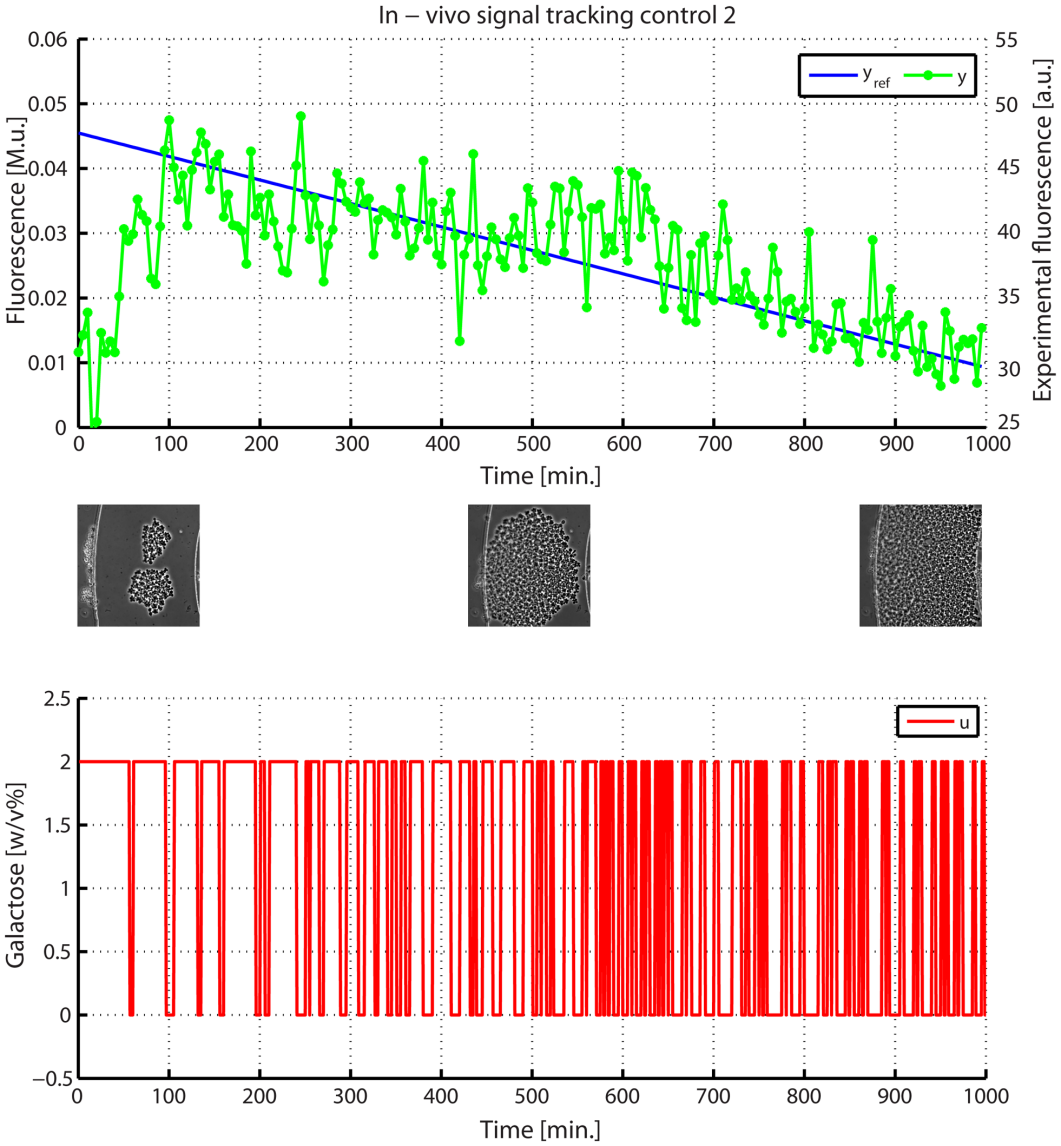
*In-vivo* signal tracking control experiment on IRMA. (Top panel) the desired (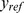 in blue) and experimentally quantified GFP (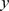 in green) are shown for the whole duration of the experiment (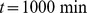). (Bottom panel) the input signal 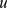 computed by the control algorithm is shown in red. (Insets) Images taken during the experiment show the growing yeast population at the beginning, at the half and at the end of the experiment.

We also performed a second signal tracking experiment where the cells, after a calibration phase, were forced to increase the expression of Gfp at the 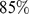 of the maximum value measured during the calibration and then to follow a linearly decreasing control reference for 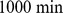 (Figure 9).

Also in this case the control task was achieved, as shown Figure 9. As before, the controller was able to keep the average of the fluorescence close to the reference signal thus the control error remained bounded for the entire experiment (Figure S22), although the number of cells was exponentially increasing (Figure S23).

These results confirm that our control platform can be applied also to control protein expression in a complex gene network.

## Discussion

The experimental results described here convincingly demonstrate that the expression of a protein can be controlled in vivo in real-time, using an inducer molecule acting directly or indirectly on protein expression, by applying principles drawn from classical control theory, and without requiring detailed quantitative knowledge of the process to be controlled, at least in the case of set-point regulation.

An experimental control platform, sharing some similarity with our work, was presented as this manuscript was under review [13]. Differently from our approach, the control scheme proposed by the authors enabled regulation of a reporter protein expression from the Hog1 promoter using osmotic pressure as a control input. The authors implemented a model predictive control scheme which relied on a pre-existing quantitative model of the Hog1 promoter to be controlled, which may not always be available when applying the scheme to a different promoter. Since the authors, for their control scheme, exploited the osmolarity pathway, which shows adaptation, they needed to develop a model-based control approach to predict the system's behaviour.

One of the advantages of our control scheme is that it can use as input any molecule and thus it may be easily transferred to the control of any other endogenous promoter, or gene network, whose dynamics can be elicited by external molecules and for which a measurable estimate of the output is available. Another useful feature is the use of a PI controller that requires minimal knowledge of the model of the system to be controlled. This generality, however, comes at a price: first, if the biological system to be controlled exhibits adaptation, or strong non-linear behaviors such as hysteresis, the PI controller is likely to fail, and a model-based control approach may be required [13], [27] (e.g. lack of controllability as investigated in Liu et al. [28]); second, the need to construct a fusion protein with a fluorescent reporter, may disrupt the physiological function of the protein.

In addition to providing an innovative platform to control protein expression in a completely automatic fashion, our results show also that binary digital pulses of an inducer molecule can be encoded and interpreted by the cell population to produce an “analog” response, i.e. a triangular wave of protein expression, or constant level of the protein.

Digital-to-analog and analog-to-digital conversion are key features of signaling pathways. Gradients of extracellular stimuli are converted into an all-or-none responses by signaling pathways [29]. These digital responses, in turn, are decoded by the cells to generate analog time-varying transcriptional responses (digital-to-analog conversion). Here we show that this core mechanism can be exploited by artificial control systems to modify at will gene and protein expression.

In this context, we wish to emphasize that, while we focused on a pulse width modulation scheme alternative strategies can indeed be devised (e.g. Pulse Amplitude or Pulse Frequency Modulation).

The control quality obtained by our control scheme is remarkably good in the case of the Gal1 endogenous promoter, but it may seem unsatisfying in the case of the IRMA network when compared to classic control engineering approaches applied to engineering systems and devices. This is the first attempt to control gene expression in a complex network using feedback control in a noisy biological system. Indeed, the presence of cell-to-cell variability is one of the key obstacles when implementing control strategies for living systems. This is why here we aimed at controlling the average fluorescence level of the cell population, which is shown to converge towards the desired value. Moreover, the control scheme keeps biological noise from increasing and at a physiological level as estimated by the CV. Interestingly, in a related work [13] reporting the result of a control strategy applied to a simpler gene circuit (a single promoter), the observed cell-to-cell variability is comparable to ours, demonstrating the inevitability of biological noise, and the challenges laying ahead for controlling gene expression in living cells.

The microfluidics-based control strategy we developed enables control experiments using small volumes of reagents with minimal perturbations to the cells. It can be easily implemented with limited costs to fine tune the expression of a protein of interest from an endogenous promoter with minimal intervention (i.e. introduction of a fluorescent reporter gene).

We believe that experimental biologists will find new and clever ways to apply our approach to study trafficking or signalling pathways and the endogenous control mechanisms of a cell.

Indeed the ability to simply overexpress a protein has led to innumerable new discoveries, and with our work we are providing a new ability which could be beneficial to many.

## Supporting Information

Figure S1
**IRMA hybrid model.** A hybrid model featuring two distinct vector fields (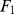 and 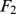) has been derived from the model presented in [17]. As long as Glucose is administered (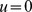) 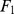 is activated, while the system switches to 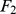 as soon as Galactose is added to the medium to reflect the inner dynamics of the synthetic circuits to be controlled.(TIF)

Figure S2
**IRMA control scheme.** The upper block scheme represents the control algorithm. The lower block magnifies the Predictor block referred to as 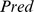 in the previous schematic. The 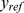 signal sets the desired output 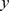 for the controlled system 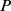. The prediction block (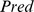) uses the input 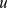 and output 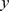 related to the actual plant 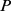 to compute an anticipated version of the output obtained by simulating the response 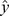 of mathematical model of 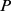 in which 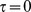. This signal is immediately used to assess the effectiveness of the control action by feeding it back to the first comparator that computes the error 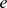 made by the system. Moreover, the actual output 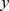 of the plant, is compared with a delayed version of the 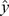 signal (as effect of the 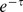 block contribution) to account for discrepancies between the predicted (via IRMA's model 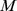) and real plant behavior. A low-pass filter meant to suppress high-frequency noise is applied to the resulting signal to obtain (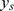) that is finally fed back to the comparator that will subtract it from 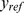 so as to obtain the control error 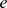.(TIF)

Figure S3
**Cohen-Coon approximation for IRMA.** In order to design a suitable PI controller we estimated three parameters, namely 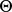, 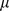 and d (as referenced in [30]) from the step response profile of the IRMA nonlinear model in equation 1–5. The solid blue line represents the response of our gene network (Cbf1p being the output) to the addition of Galactose to the growth media at 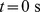 while the dashed blue line shows the same information for the time delayed linear system identified with the method in [30].(TIF)

Figure S4
**Finite State Automaton implementing the control algorithm in Figure S2.** In the initial state, state 0, the calibration is carried out as previously described. The system cycles on this state until the initialization is completed and then moves to state 1. At this point given the error 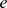, the PI - PWM block is simulated to compute the control input 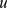. In state 2 the model prediction is calculated given 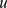; the input is then applied to the physical system by means of hydrostatic pressure modulation in step 3 (the correct amounts of Galactose/Raffinose and Glucose are provided at the end of this step). In state 4 the delayed version of computed output is calculated; during state 5, the presence of a new image is verified, and the image processing algorithm is run in order to obtain the system output measure. Given this it is possible to calculate 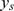 and the error 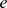 for the next control iteration. The algorithm then moves to state 1 for a new control iteration to start.(TIF)

Figure S5
***In-silico*** **prediction-based signal tracking control of IRMA.** The predictor-based algorithm is applied to control the dynamical model of IRMA to a time varying reference signal (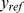, in blue); the computed control input (higher state standing for Galactose and lower state meaning Glucose providing) is represented in red (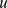). The good overlap between the reference signal and the simulated Cbf1 time evolution (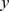) provides evidence for the robustness of the designed control scheme in two cases: (top panel) with no delay (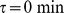) and with 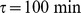 (bottom panel).(TIF)

Figure S6
***In-silico*** **prediction-based set point control of IRMA.** The predictor-based algorithm is applied to control the dynamical model of IRMA to a constant reference signal (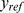 in blue). The set point is calculated as the 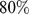 of the maximum value for the simulated Cbf1 time evolution evaluated until 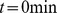. The control input (computed after time 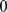 where higher state standing for Galactose and lower state meaning Glucose providing) is represented in red (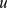). The simulation was performed with the dynamical model without delay (top panel) or with a delay 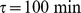 (bottom panel). In both cases, the control action is able to guarantee good dynamical performances of the system, indeed the simulated Cbf1 time evolution (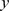 in green) tightly matches the reference signal.(TIF)

Figure S7
***In-silico*** **PI/PWM signal tracking control of IRMA.** The PI/PWM control algorithm is applied to control the dynamical model of IRMA to a time varying reference signal (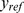, in blue); the computed control input (high level: Galactose; low level: Glucose) is shown in red (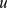); the Cbf1 time evolution is shown in green (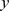). When the control is applied to the model without the delay, the control output (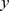) follows the reference signal (top panel); whereas the PI - PWM is not able to achieve the control objective for the model with the delay (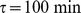) (bottom panel).(TIF)

Figure S8
***In-silico*** **PI/PWM set point control of IRMA.** The PI/PWM control algorithm is applied to control the dynamical model of IRMA to a constant reference signal (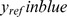). The set point is equal to 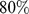 of the maximum value for the simulated Cbf1 time evolution evaluated until 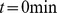. The control input, computed after time 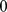, is shown in red (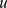 high level: Galactose; low level: Glucose). The simulation was performed with the dynamical model without delay (top panel) or with a delay 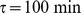 (bottom panel). When the control is applied to the model without delay, the control output (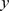) follows the reference signal (top panel); on the contrary, the PI - PWM is not able to achieve the control objective for the model with the delay (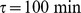) (bottom panel).(TIF)

Figure S9 **Image processing.** The algorithm applies Otsu thresholding to binarize the grey scale phase contrast image (A). Convex hulls (B) are then used to limit the application of the Circular Hought Transform to find cells' centers and edges (C).
(TIF)

Figure S10
***In-vivo*** **set point control experiment no. 1 for the** ***GAL1*** **promoter - fluorescence standard deviation.** By using the off-line analysis described in the text, it is possible to compute the standard deviation of the fluorescence for each frame acquired during the control experiment. The desired amount of protein (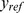 in blue), the quantified GFP (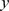 green line) and the standard deviation's upper and lower bounds (thin green lines) are shown; the control error (top pane in black) is computed as the difference between the feedback signal and the control reference. The input signal 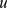 computed by the control algorithm is shown in red (bottom panel).(TIF)

Figure S11
***In-vivo*** **set point control experiment no. 2 for the** ***GAL1*** **promoter - fluorescence standard deviation.** By using the off-line analysis described in the text, it is possible to compute the standard deviation of the fluorescence for each frame acquired during the control experiment. The desired amount of protein (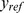 in blue), the quantified GFP (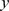 green line) and the standard deviation's upper and lower bounds (thin green lines) are shown; the control error (top pane in black) is computed as the difference between the feedback signal and the control reference. The input signal 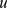 computed by the control algorithm is shown in red (bottom panel).(TIF)

Figure S12
***In-vivo*** **set point control experiment no. 3 for the** ***GAL1*** **promoter - fluorescence standard deviation.** By using the off-line analysis described in the text, it is possible to compute the standard deviation of the fluorescence for each frame acquired during the control experiment. The desired amount of protein (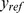 in blue), the quantified GFP (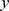 green line) and the standard deviation's upper and lower bounds (thin green lines) are shown; the control error (top pane in black) is computed as the difference between the feedback signal and the control reference. The input signal 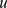 computed by the control algorithm is shown in red (bottom panel).(TIF)

Figure S13
***In-vivo*** **set point control experiment no. 4 for the** ***GAL1*** **promoter - fluorescence standard deviation.** By using the off-line analysis described in the text, it is possible to compute the standard deviation of the fluorescence for each frame acquired during the control experiment. The desired amount of protein (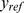 in blue), the quantified GFP (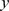 green line) and the standard deviation's upper and lower bounds (thin green lines) are shown; the control error (top pane in black) is computed as the difference between the feedback signal and the control reference. The input signal 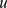 computed by the control algorithm is shown in red (bottom panel).(TIF)

Figure S14 ***In-vivo*** **set point control experiments** ***GAL1*** **promoter - cell count and coefficient of variation.** (A-D) For each of the experiments of Supplementary Figures S10, S11, S12 and S13, the number of cells (top) and the coefficient of variation (bottom) are shown.
(TIF)

Figure S15
***In-vivo*** **set point control experiment for the IRMA network - fluorescence standard deviation.** By using the off-line analysis described in the text it is possible to calculate the standard deviation of the fluorescence for each frame acquired during the control. The desired amount of protein (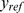 in blue), the quantified GFP (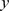 green line), the standard deviation's upper and lower bounds (thin green lines) and the control error 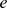 in black are shown; mean 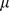, variance 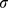 and coefficient of variation 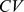 of the control error are also shown; the p-value was computed as described in the Supplementary Information text (top panel). The input signal 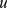 computed by the control algorithm is shown in red (bottom panel).(TIF)

Figure S16 ***In-vivo*** **signal tracking control experiment for the IRMA network - cell count and coefficient of variation.** For the experiment of Supplementary Figure S15, the number of cell (top panel) and the coefficient of variation (bottom panel) are shown.
(TIF)

Figure S17 **Response to a sustained galactose input for the IRMA network.** Green line: fluorescence measured when the cells are treated with galactose for the whole experiment; light green line: fluorescence measured during the in-vivo set point control experiment (Figure 7 - main text); black line: the control reference of the set-point control experiment (Figure 7 - main text); red line: the sustained galactose input provided to the cells population; light red: the input calculated automatically by the control algorithm and used to regulate the production of GFP to the desired level in in-vivo set point control experiment (Figure 7 - main text).
(TIF)

Figure S18 **Response to a sustained galactose input for the IRMA network.** Green line: fluorescence measured when the cells are treated with galactose for the whole experiment; blue line: the control reference of the set-point control experiment (Figure 7 - main text) (Top panel).(Bottom panel) red line: the sustained galactose input administered to cells. The normalised root mean square error (NRMSE) of the deviation between the blue and the green signal has been reported to be equal to 0.33.
(TIF)

Figure S19
***In-vivo*** **signal tracking control experiment for the IRMA network - fluorescence standard deviation.** By using the off-line analysis described in the text it is possible to calculate the standard deviation of the fluorescence for each frame acquired during the control. The desired amount of protein (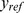 in blue), the quantified GFP (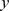 green line) and its upper and lower bound of the standard deviation (thin green lines) are plotted (top panel). The input signal 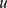 computed by the control algorithm is shown in red (bottom panel).(TIF)

Figure S20 ***In-vivo*** **signal tracking control experiment for the IRMA network - cell count and coefficient of variation.** For the experiment of Figure S19, the number of cell (top panel) and the coefficient of variation (bottom panel) are plotted.
(TIF)

Figure S21
**Internal signals of the control experiment in** **Fig. 8** **(main text).** Time evolution of the most relevant signals in the control loop are shown. In particular the Galactose concentration in the medium (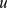) provided to the cells has been plotted in red, while the output of the delay-free model (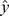) and its delayed version (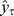) are shown in green and violet respectively. The error signal 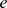 (black) calculated as the difference between 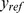 and 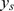 (cyan) is also depicted; mean 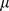, variance 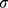 and coefficient of variation 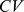 of the control error are also shown.(TIF)

Figure S22
***In-vivo*** **signal tracking control experiment 2 for the IRMA network - fluorescence standard deviation.** By using the off-line analysis described in the text it is possible to calculate the standard deviation of the fluorescence for each frame acquired during the control. The desired amount of protein (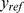 in blue), the quantified GFP (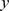 green line) and its upper and lower bound of the standard deviation (thin green lines) are plotted; the control error calculated as the difference between the feedback signal and the control reference is shown in black (top panel). The input signal 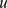 computed by the control algorithm is shown in red (bottom panel).(TIF)

Figure S23 ***In-vivo*** **signal tracking control experiment 2 for the IRMA network - cell count and coefficient of variation.** For the experiment of Figure S22, the number of cell (top panel) and the coefficient of variation(bottom panel) are plotted.
(TIF)

Figure S24 **Calibration phase.** The calibration data have been reported for the experiment in Fig. 8. The simulated (blue) and quantified (green) Gfp evolution have been used to relate fluorescence data to model predictions (model units).
(TIF)

Figure S25 **IRMA switch off experiment.** Top panel: the green signals represent the measured fluorescence during *in-vivo* switch - off experiments, the blue signal is the result of *in-silico* switch off experiment using the dynamical model of IRMA (all the experimental signals are rescaled to the model range). Bottom panel: the input used to perform the experiment; cells have been fed for 180 minutes with galactose (ON signal, 1 for the mathematical model) and for 420 minutes with glucose (OFF signal, 0 for the mathematical model).
(TIF)

Text S1 **Supplementary information text.** All the additional details concerning materials and methods of the present work are here reported.
(PDF)

Video S1
**Movie of the experiment in** **Figure 8** **.** (Top left panel) Yeast cell fluorescence during the control experiment; (top right panel) cell count; (bottom left panel) desired (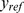 in blue) experimentally quantified GFP fluorescence (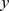 in green) and input (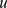 in black) calculated by the control algorithm are shown for the whole duration of the experiment; (bottom right panel) histogram of the cell fluorescence distribution.(MPG)

Video S2
**Movie of the experiment in** **Figure 9** **.** (Top left panel) Yeast cell fluorescence during the control experiment; (top right panel) cell count; (bottom left panel) desired (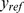 in blue) experimentally quantified GFP fluorescence (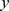 in green) and input (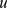 in black) calculated by the control algorithm are shown for the whole duration of the experiment; (bottom right panel) histogram of the cell fluorescence distribution.(MPG)
