# Supplementary figures and images for: In-Vivo Real-Time Control of Protein Expression from Endogenous and Synthetic Gene Networks
Source: PLoS Comput Biol. 2014 May 15;10(5):e1003625. doi: 10.1371/journal.pcbi.1003625 (PMC4022480; doi:10.1371/journal.pcbi.1003625)

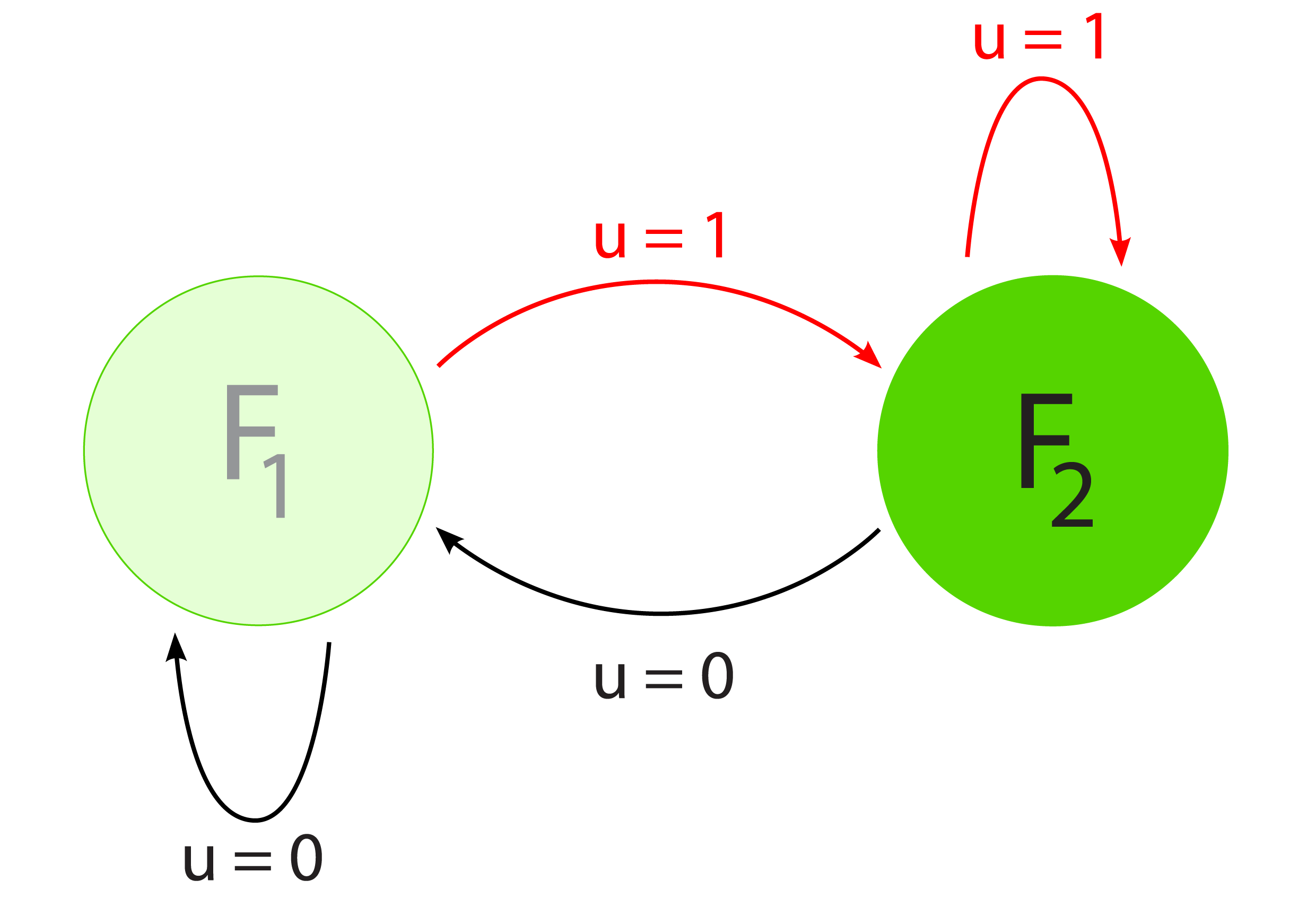

Supplement: Figure S1 — IRMA hybrid model. A hybrid model featuring two distinct vector fields ( and ) has been derived from the model presented in [17]. As long as Glucose is administered () is activated, while the system switches to as soon as Galactose is added to the medium to reflect the inner dynamics of the synthetic circuits to be controlled. (TIF) [file pcbi.1003625.s001.tif]

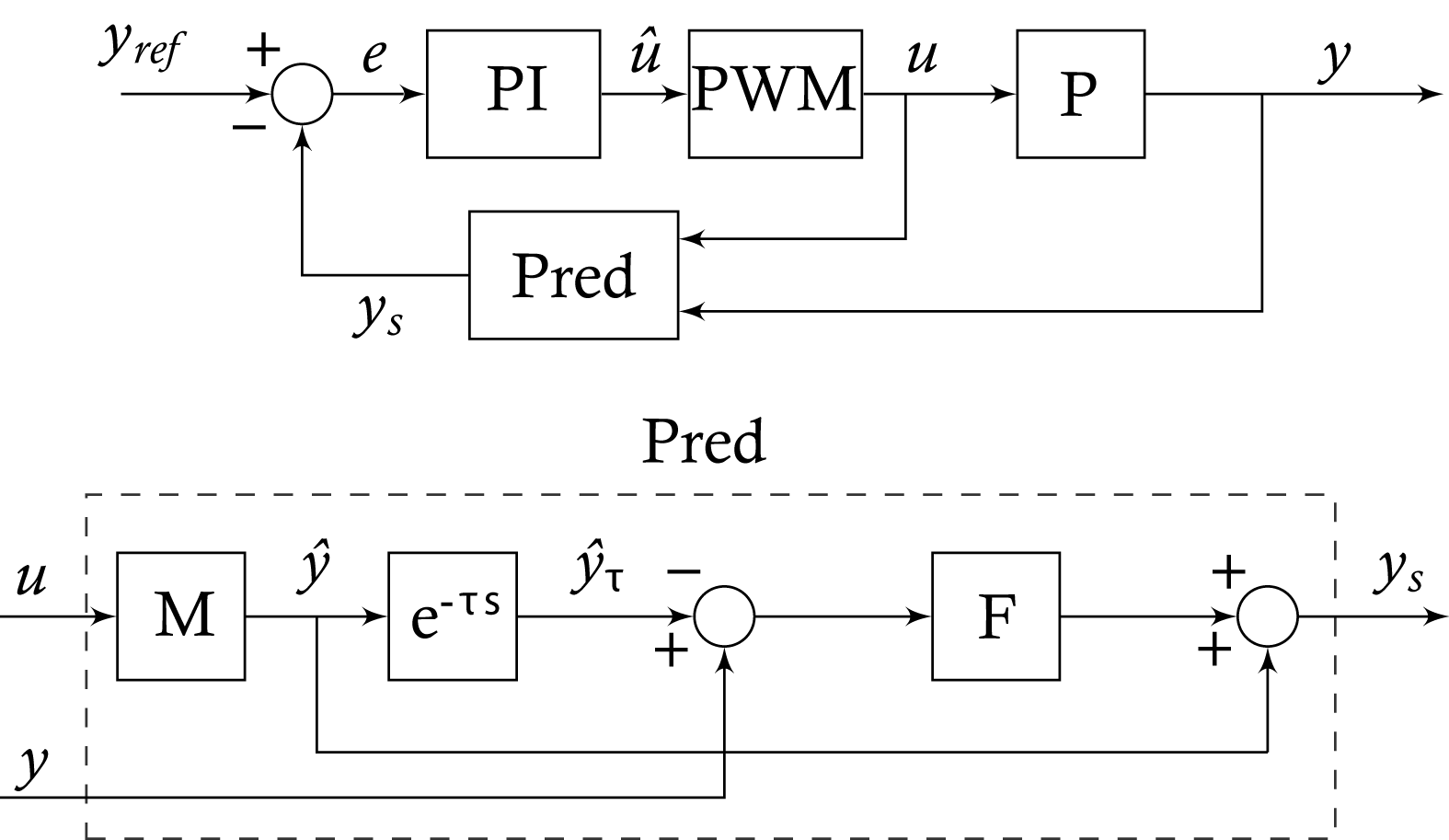

Supplement: Figure S2 — IRMA control scheme. The upper block scheme represents the control algorithm. The lower block magnifies the Predictor block referred to as in the previous schematic. The signal sets the desired output for the controlled system . The prediction block () uses the input and output related to the actual plant to compute an anticipated version of the output obtained by simulating the response of mathematical model of in which . This signal is immediately used to assess the effectiveness of the control action by feeding it back to the first comparator that computes the error made by the system. Moreover, the actual output of the plant, is compared with a delayed version of the signal (as effect of the block contribution) to account for discrepancies between the predicted (via IRMA's model ) and real plant behavior. A low-pass filter meant to suppress high-frequency noise is applied to the resulting signal to obtain () that is finally fed back to the comparator that will subtract it from so as to obtain the control error . (TIF) [file pcbi.1003625.s002.tif]

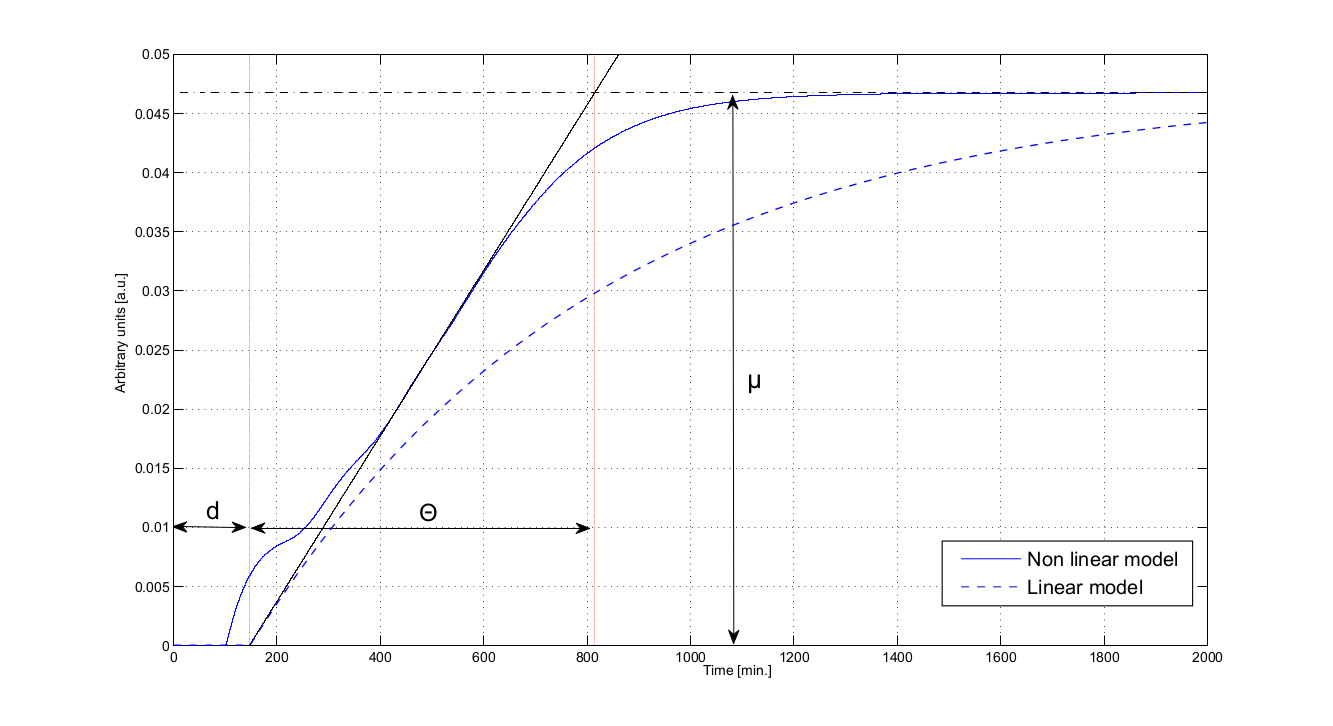

Supplement: Figure S3 — Cohen-Coon approximation for IRMA. In order to design a suitable PI controller we estimated three parameters, namely , and d (as referenced in [30]) from the step response profile of the IRMA nonlinear model in equation 1–5. The solid blue line represents the response of our gene network (Cbf1p being the output) to the addition of Galactose to the growth media at while the dashed blue line shows the same information for the time delayed linear system identified with the method in [30]. (TIF) [file pcbi.1003625.s003.tif]

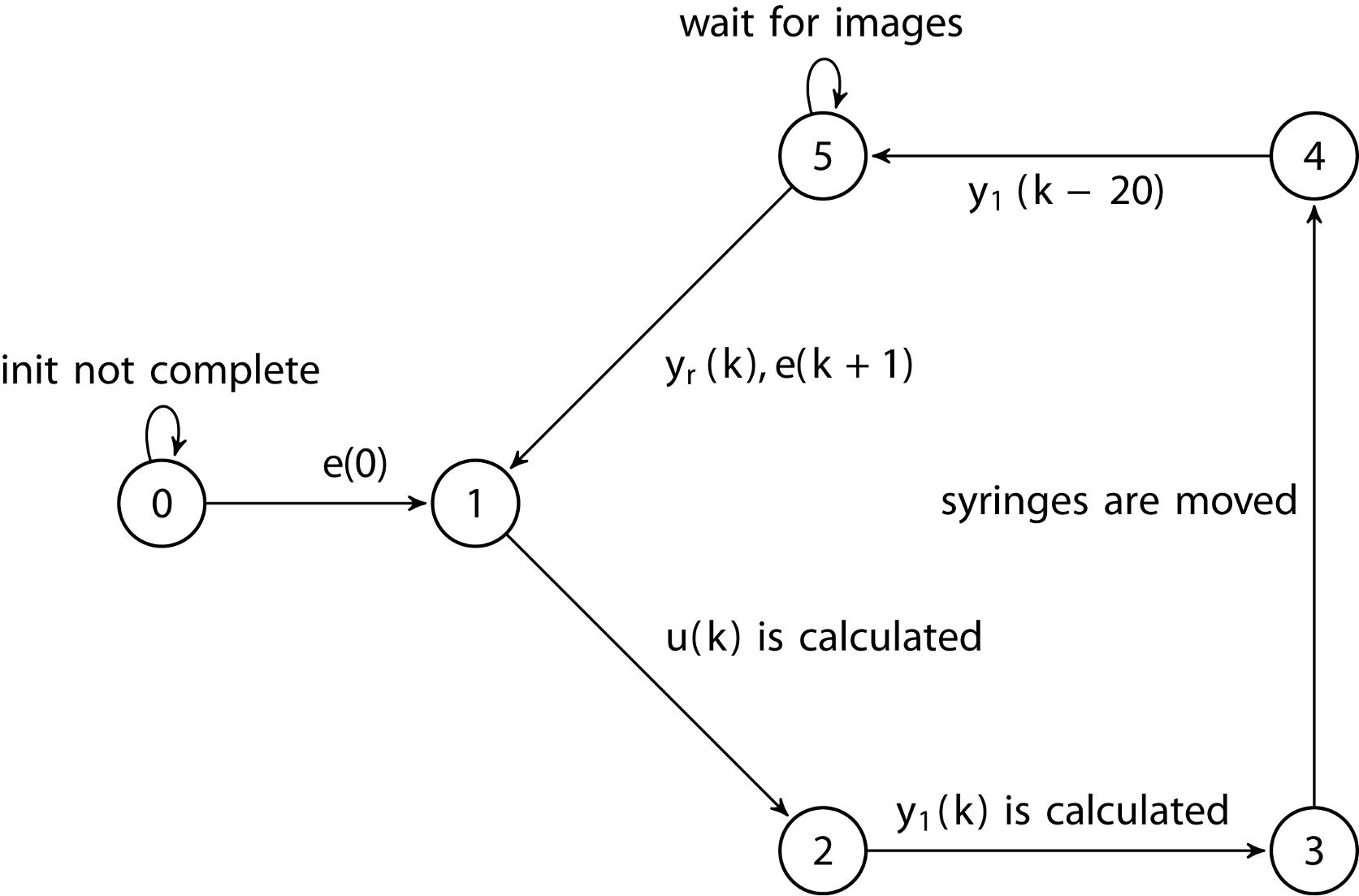

Supplement: Figure S4 — Finite State Automaton implementing the control algorithm in Figure S2. In the initial state, state 0, the calibration is carried out as previously described. The system cycles on this state until the initialization is completed and then moves to state 1. At this point given the error , the PI - PWM block is simulated to compute the control input . In state 2 the model prediction is calculated given ; the input is then applied to the physical system by means of hydrostatic pressure modulation in step 3 (the correct amounts of Galactose/Raffinose and Glucose are provided at the end of this step). In state 4 the delayed version of computed output is calculated; during state 5, the presence of a new image is verified, and the image processing algorithm is run in order to obtain the system output measure. Given this it is possible to calculate and the error for the next control iteration. The algorithm then moves to state 1 for a new control iteration to start. (TIF) [file pcbi.1003625.s004.tif]

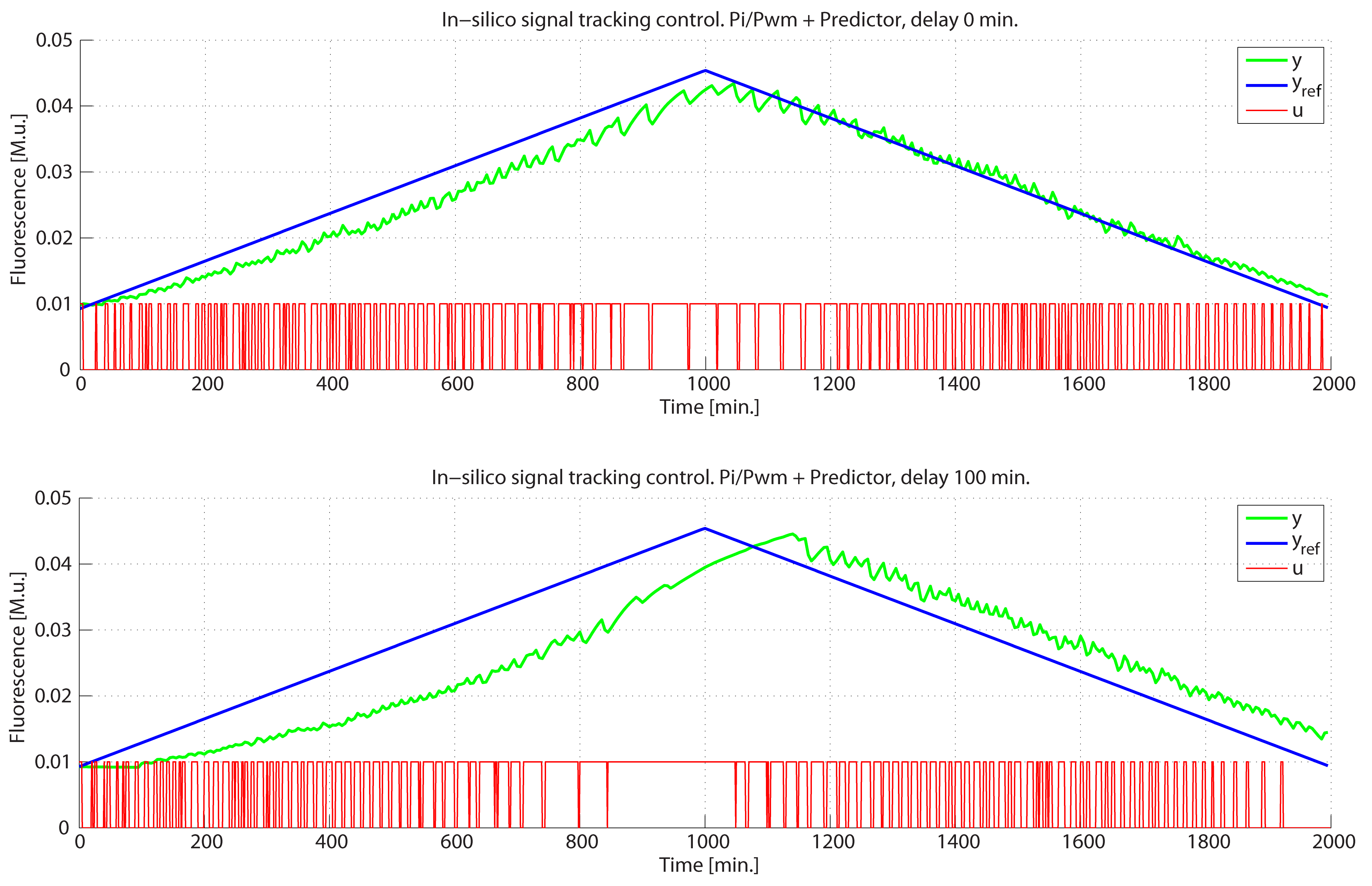

Supplement: Figure S5 — In-silico prediction-based signal tracking control of IRMA. The predictor-based algorithm is applied to control the dynamical model of IRMA to a time varying reference signal (, in blue); the computed control input (higher state standing for Galactose and lower state meaning Glucose providing) is represented in red (). The good overlap between the reference signal and the simulated Cbf1 time evolution () provides evidence for the robustness of the designed control scheme in two cases: (top panel) with no delay () and with (bottom panel). (TIF) [file pcbi.1003625.s005.tif]

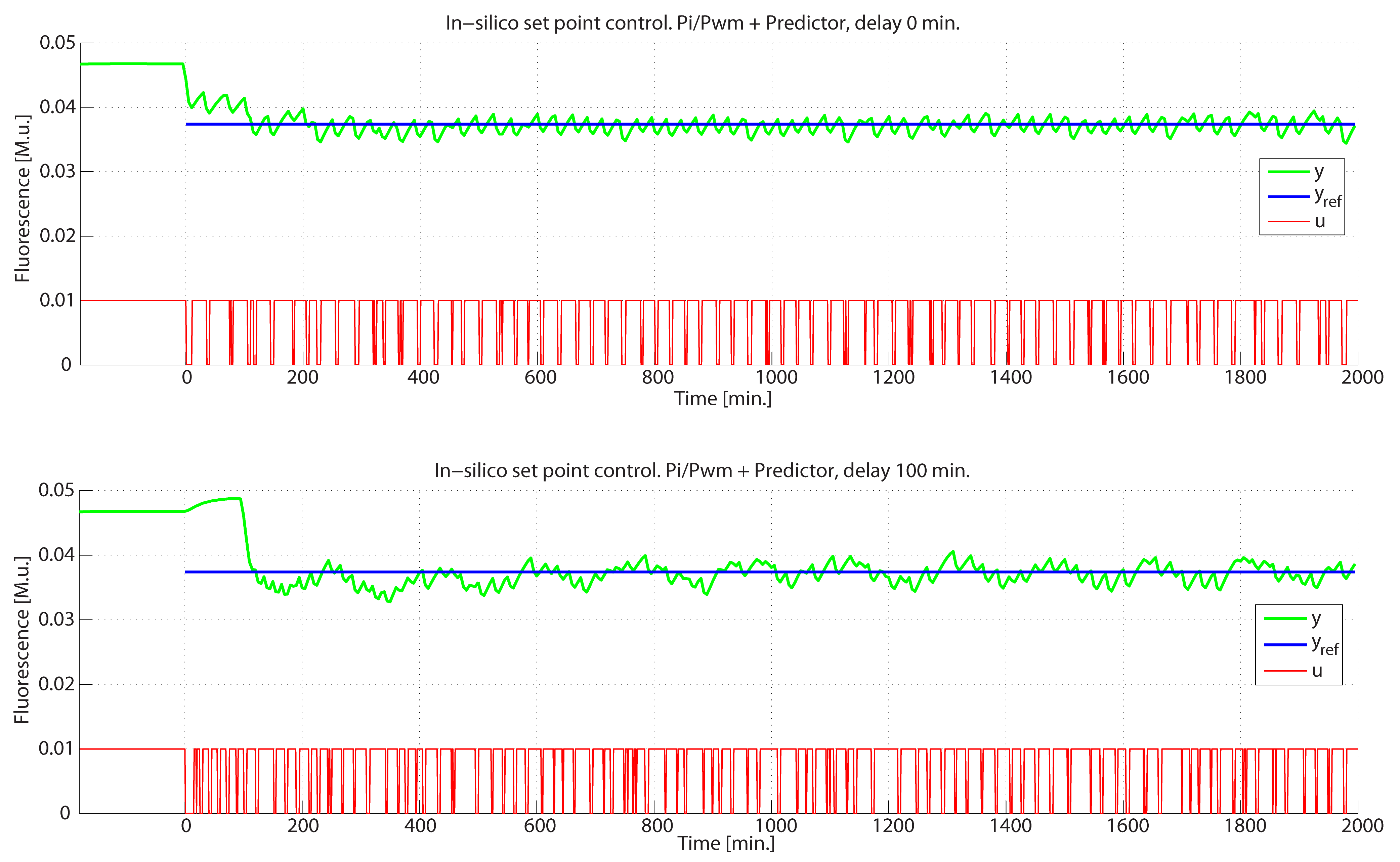

Supplement: Figure S6 — In-silico prediction-based set point control of IRMA. The predictor-based algorithm is applied to control the dynamical model of IRMA to a constant reference signal ( in blue). The set point is calculated as the of the maximum value for the simulated Cbf1 time evolution evaluated until . The control input (computed after time where higher state standing for Galactose and lower state meaning Glucose providing) is represented in red (). The simulation was performed with the dynamical model without delay (top panel) or with a delay (bottom panel). In both cases, the control action is able to guarantee good dynamical performances of the system, indeed the simulated Cbf1 time evolution ( in green) tightly matches the reference signal. (TIF) [file pcbi.1003625.s006.tif]

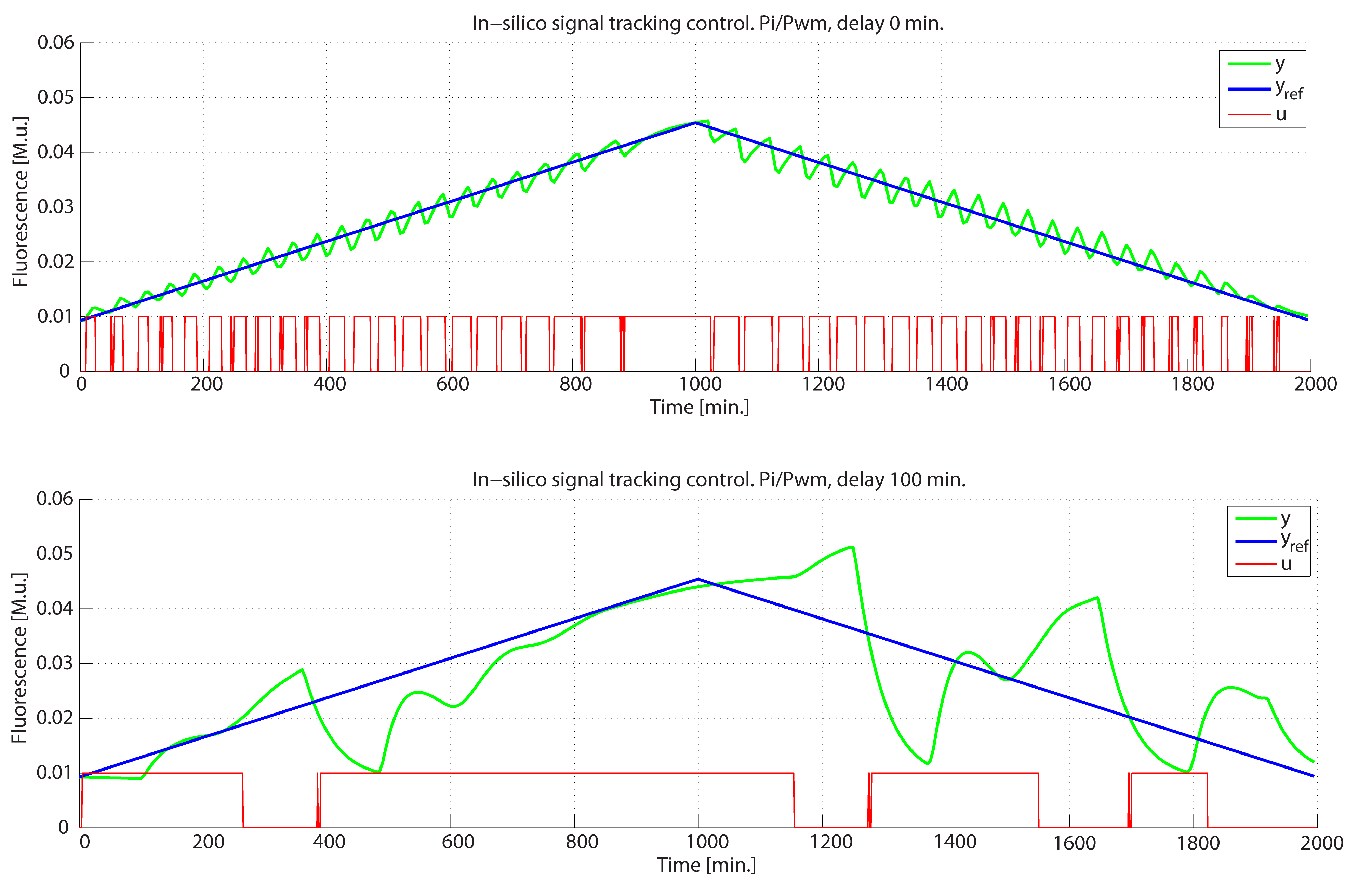

Supplement: Figure S7 — In-silico PI/PWM signal tracking control of IRMA. The PI/PWM control algorithm is applied to control the dynamical model of IRMA to a time varying reference signal (, in blue); the computed control input (high level: Galactose; low level: Glucose) is shown in red (); the Cbf1 time evolution is shown in green (). When the control is applied to the model without the delay, the control output () follows the reference signal (top panel); whereas the PI - PWM is not able to achieve the control objective for the model with the delay () (bottom panel). (TIF) [file pcbi.1003625.s007.tif]

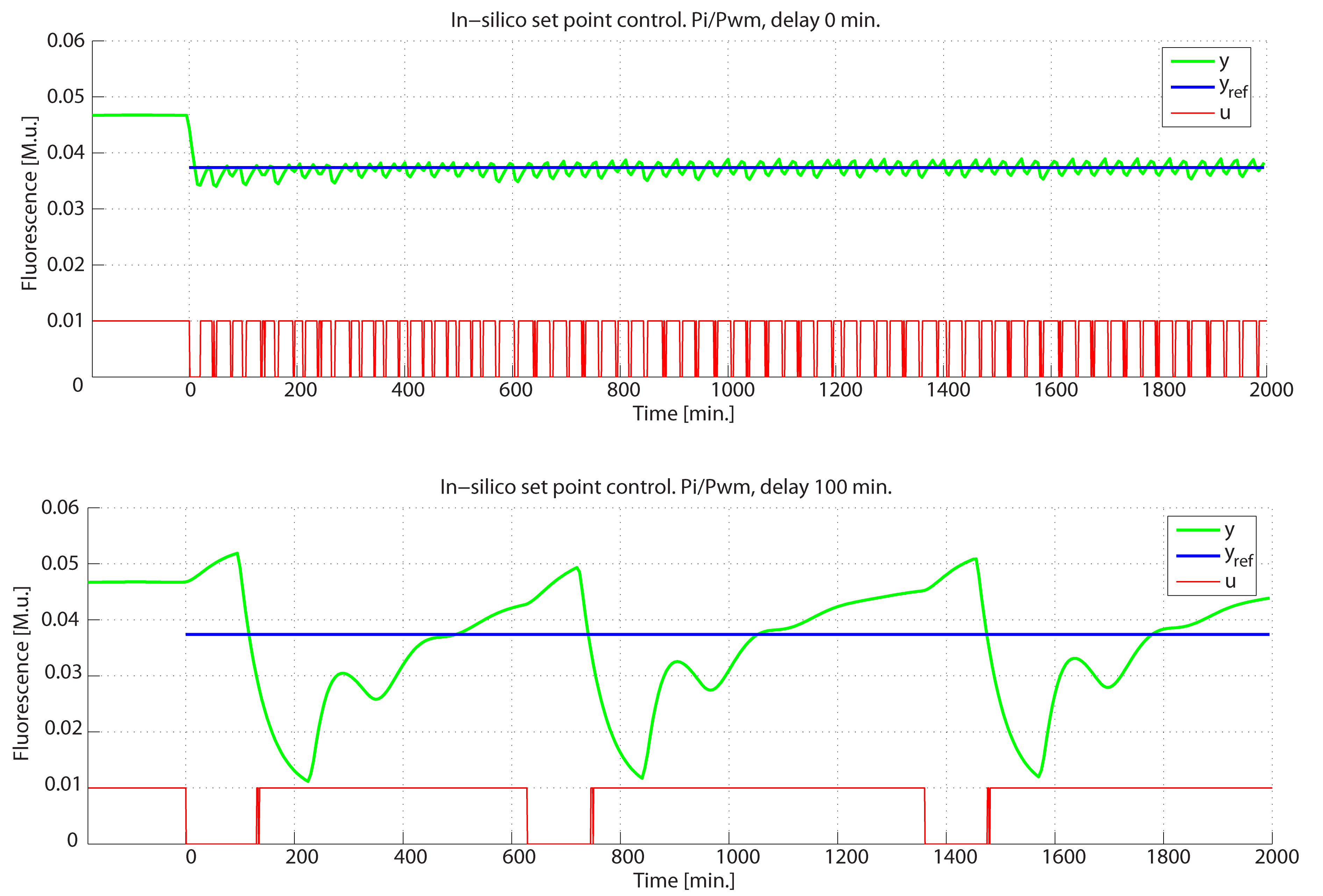

Supplement: Figure S8 — In-silico PI/PWM set point control of IRMA. The PI/PWM control algorithm is applied to control the dynamical model of IRMA to a constant reference signal (). The set point is equal to of the maximum value for the simulated Cbf1 time evolution evaluated until . The control input, computed after time , is shown in red ( high level: Galactose; low level: Glucose). The simulation was performed with the dynamical model without delay (top panel) or with a delay (bottom panel). When the control is applied to the model without delay, the control output () follows the reference signal (top panel); on the contrary, the PI - PWM is not able to achieve the control objective for the model with the delay () (bottom panel). (TIF) [file pcbi.1003625.s008.tif]

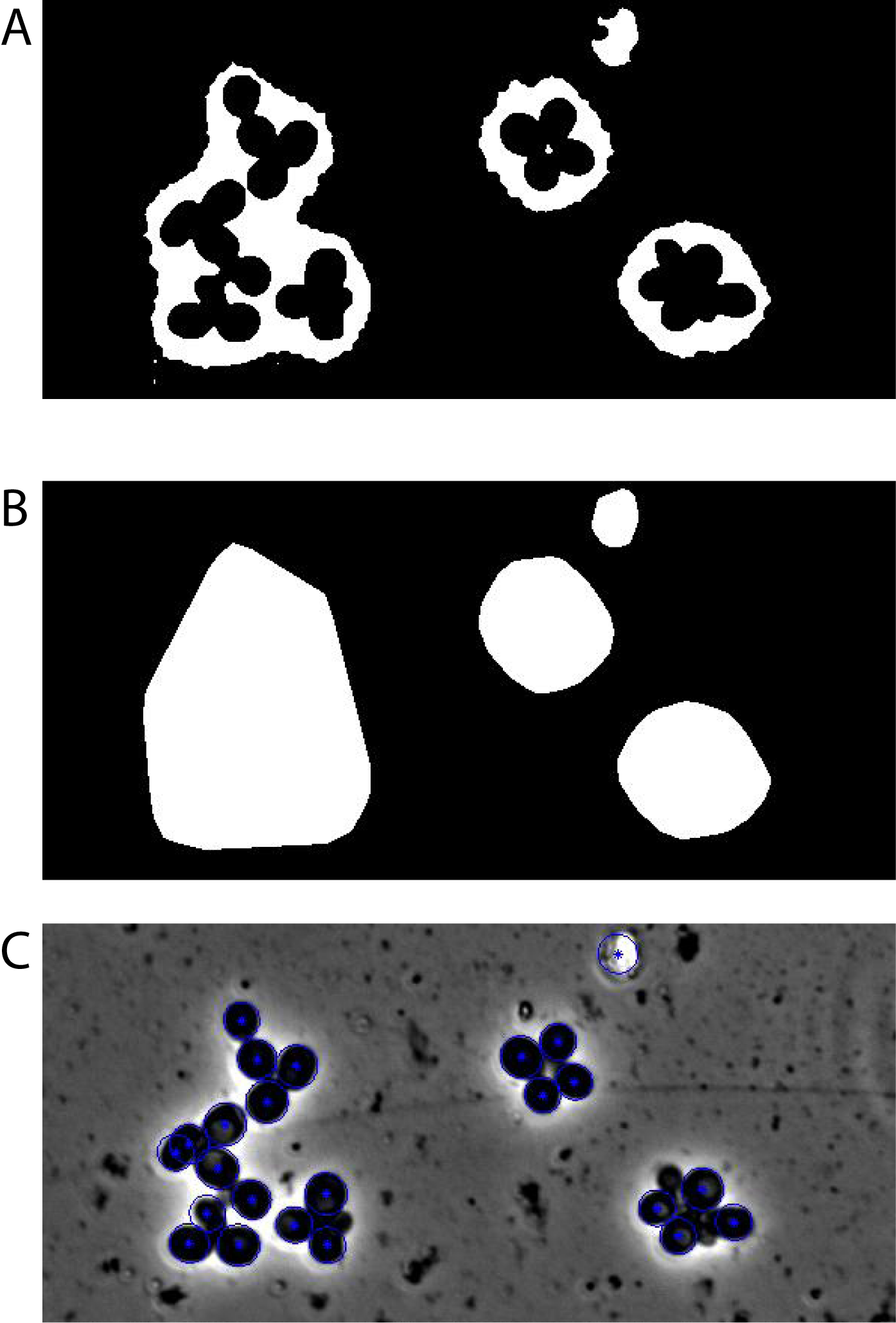

Supplement: Figure S9 — Image processing. The algorithm applies Otsu thresholding to binarize the grey scale phase contrast image (A). Convex hulls (B) are then used to limit the application of the Circular Hought Transform to find cells' centers and edges (C). (TIF) [file pcbi.1003625.s009.tif]

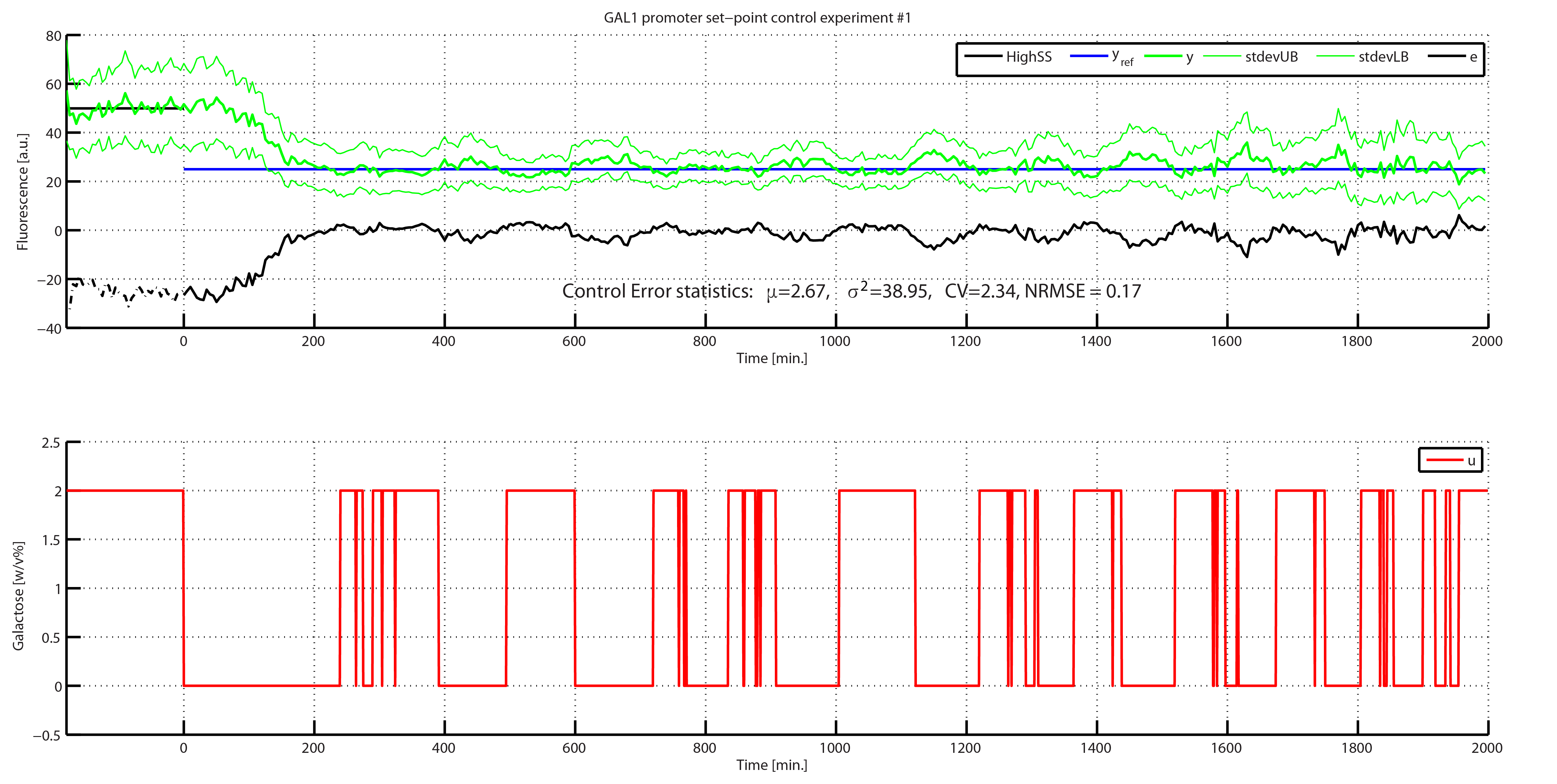

Supplement: Figure S10 — In-vivo set point control experiment no. 1 for the GAL1 promoter - fluorescence standard deviation. By using the off-line analysis described in the text, it is possible to compute the standard deviation of the fluorescence for each frame acquired during the control experiment. The desired amount of protein ( in blue), the quantified GFP ( green line) and the standard deviation's upper and lower bounds (thin green lines) are shown; the control error (top pane in black) is computed as the difference between the feedback signal and the control reference. The input signal computed by the control algorithm is shown in red (bottom panel). (TIF) [file pcbi.1003625.s010.tif]

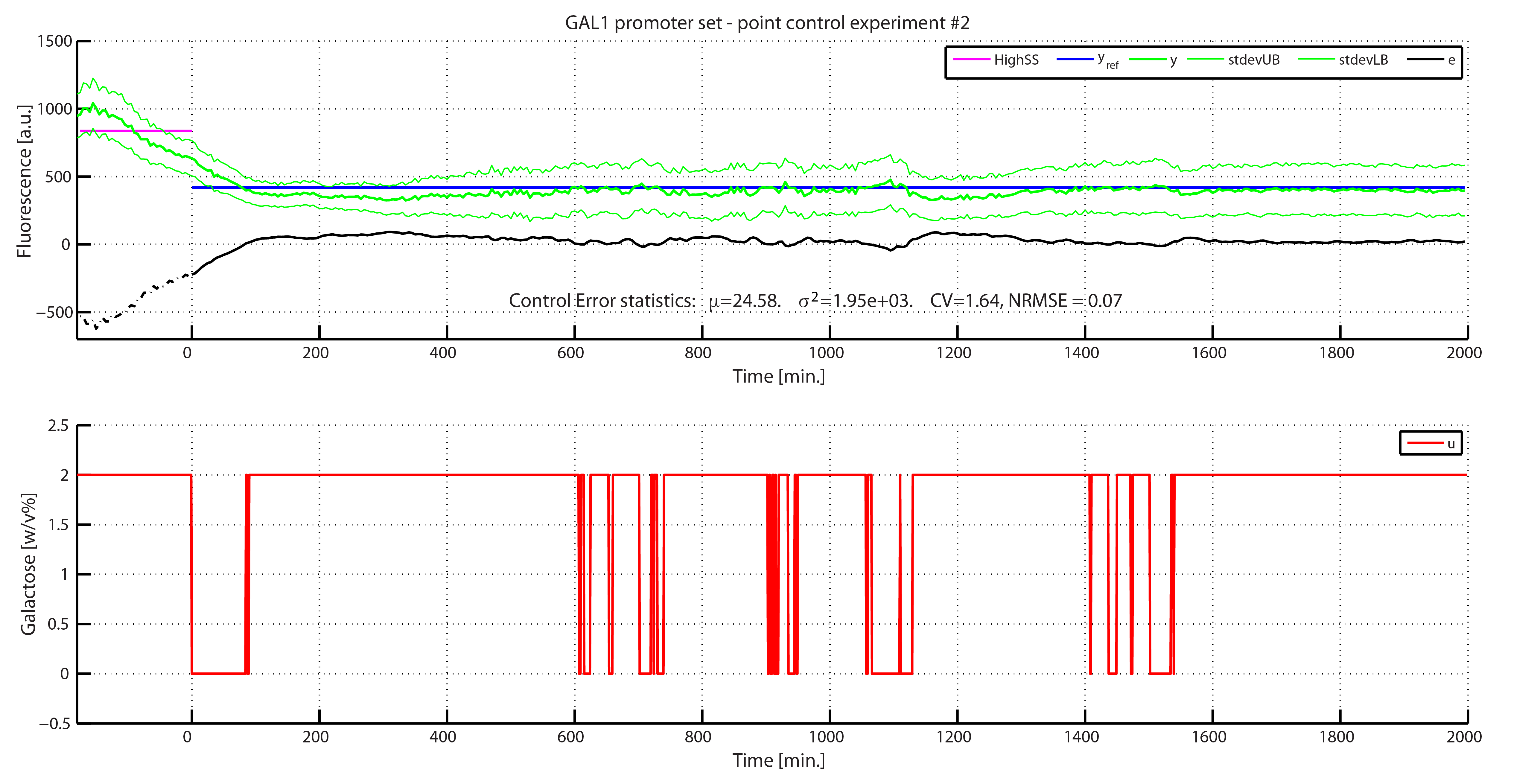

Supplement: Figure S11 — In-vivo set point control experiment no. 2 for the GAL1 promoter - fluorescence standard deviation. By using the off-line analysis described in the text, it is possible to compute the standard deviation of the fluorescence for each frame acquired during the control experiment. The desired amount of protein ( in blue), the quantified GFP ( green line) and the standard deviation's upper and lower bounds (thin green lines) are shown; the control error (top pane in black) is computed as the difference between the feedback signal and the control reference. The input signal computed by the control algorithm is shown in red (bottom panel). (TIF) [file pcbi.1003625.s011.tif]

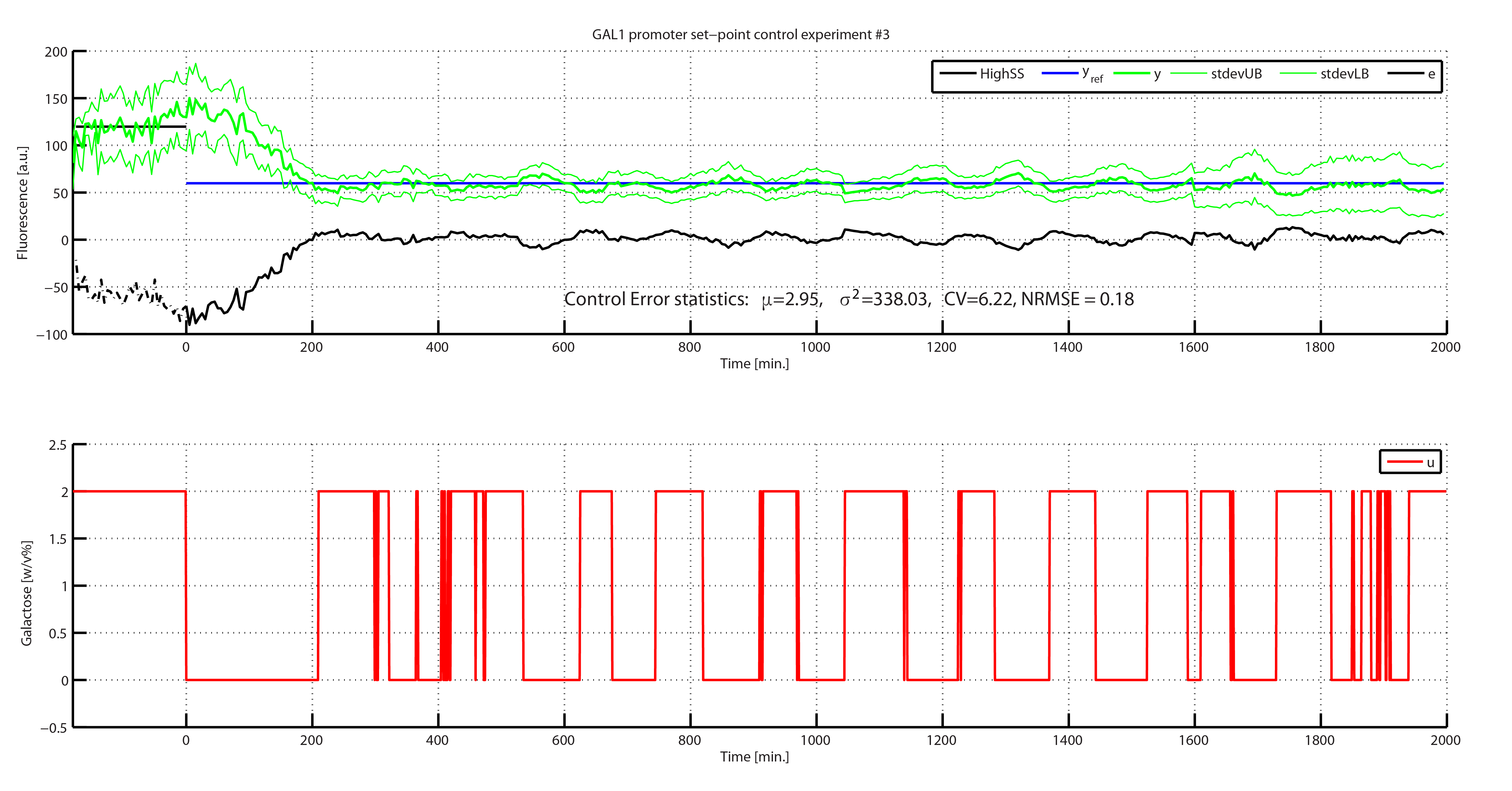

Supplement: Figure S12 — In-vivo set point control experiment no. 3 for the GAL1 promoter - fluorescence standard deviation. By using the off-line analysis described in the text, it is possible to compute the standard deviation of the fluorescence for each frame acquired during the control experiment. The desired amount of protein ( in blue), the quantified GFP ( green line) and the standard deviation's upper and lower bounds (thin green lines) are shown; the control error (top pane in black) is computed as the difference between the feedback signal and the control reference. The input signal computed by the control algorithm is shown in red (bottom panel). (TIF) [file pcbi.1003625.s012.tif]

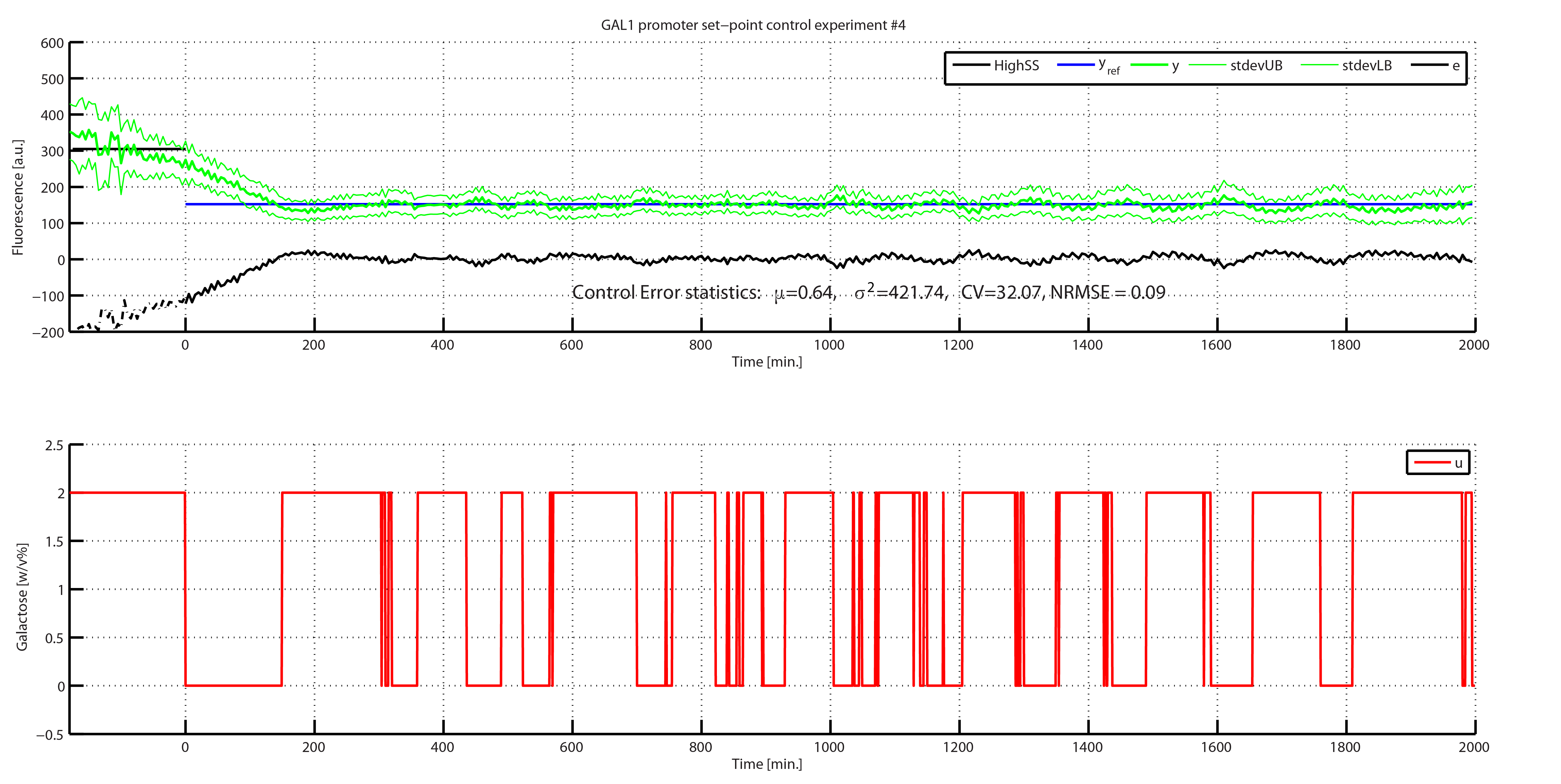

Supplement: Figure S13 — In-vivo set point control experiment no. 4 for the GAL1 promoter - fluorescence standard deviation. By using the off-line analysis described in the text, it is possible to compute the standard deviation of the fluorescence for each frame acquired during the control experiment. The desired amount of protein ( in blue), the quantified GFP ( green line) and the standard deviation's upper and lower bounds (thin green lines) are shown; the control error (top pane in black) is computed as the difference between the feedback signal and the control reference. The input signal computed by the control algorithm is shown in red (bottom panel). (TIF) [file pcbi.1003625.s013.tif]

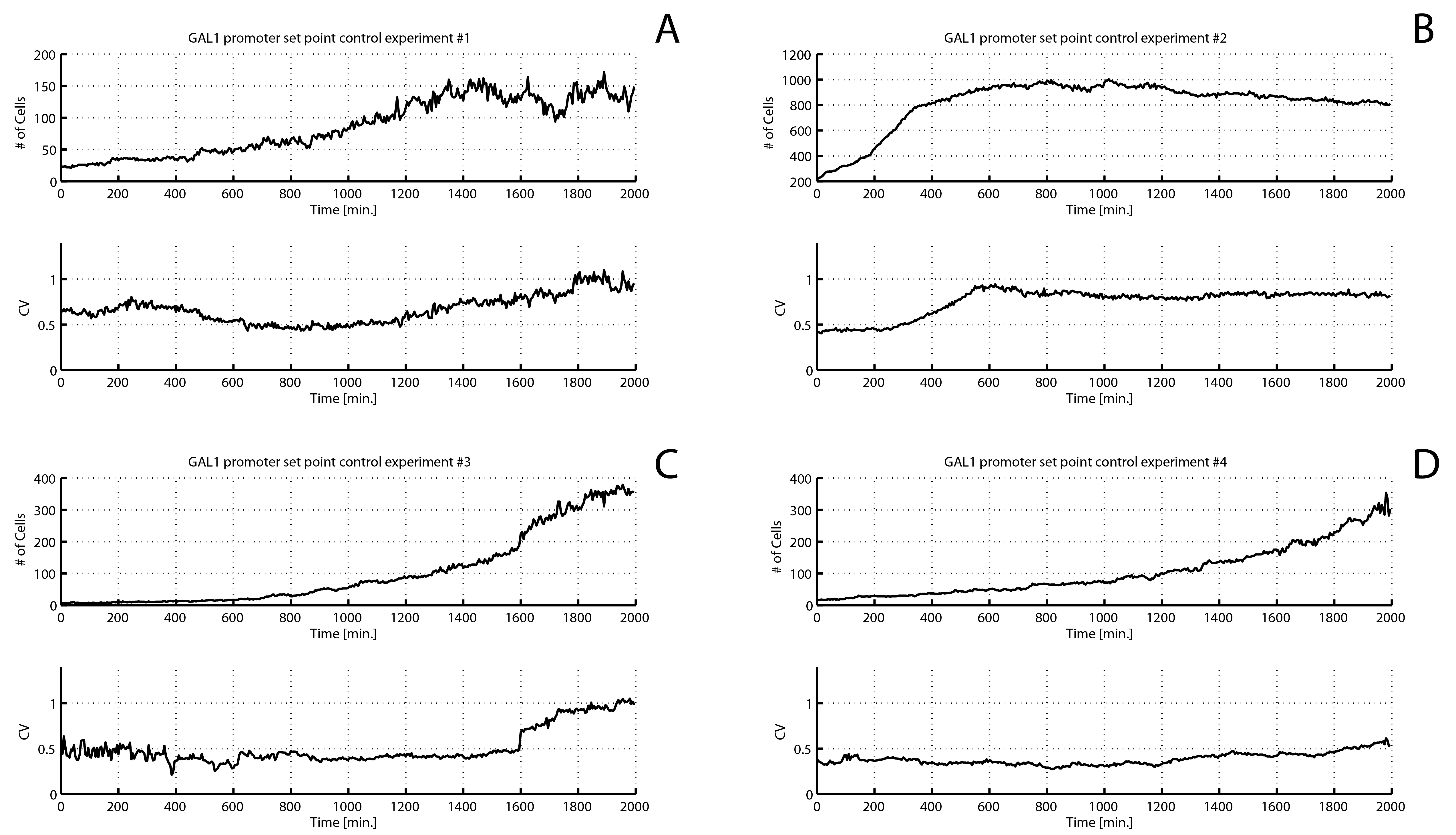

Supplement: Figure S14 — In-vivo set point control experiments GAL1 promoter - cell count and coefficient of variation. (A-D) For each of the experiments of Supplementary Figures S10, S11, S12 and S13, the number of cells (top) and the coefficient of variation (bottom) are shown. (TIF) [file pcbi.1003625.s014.tif]

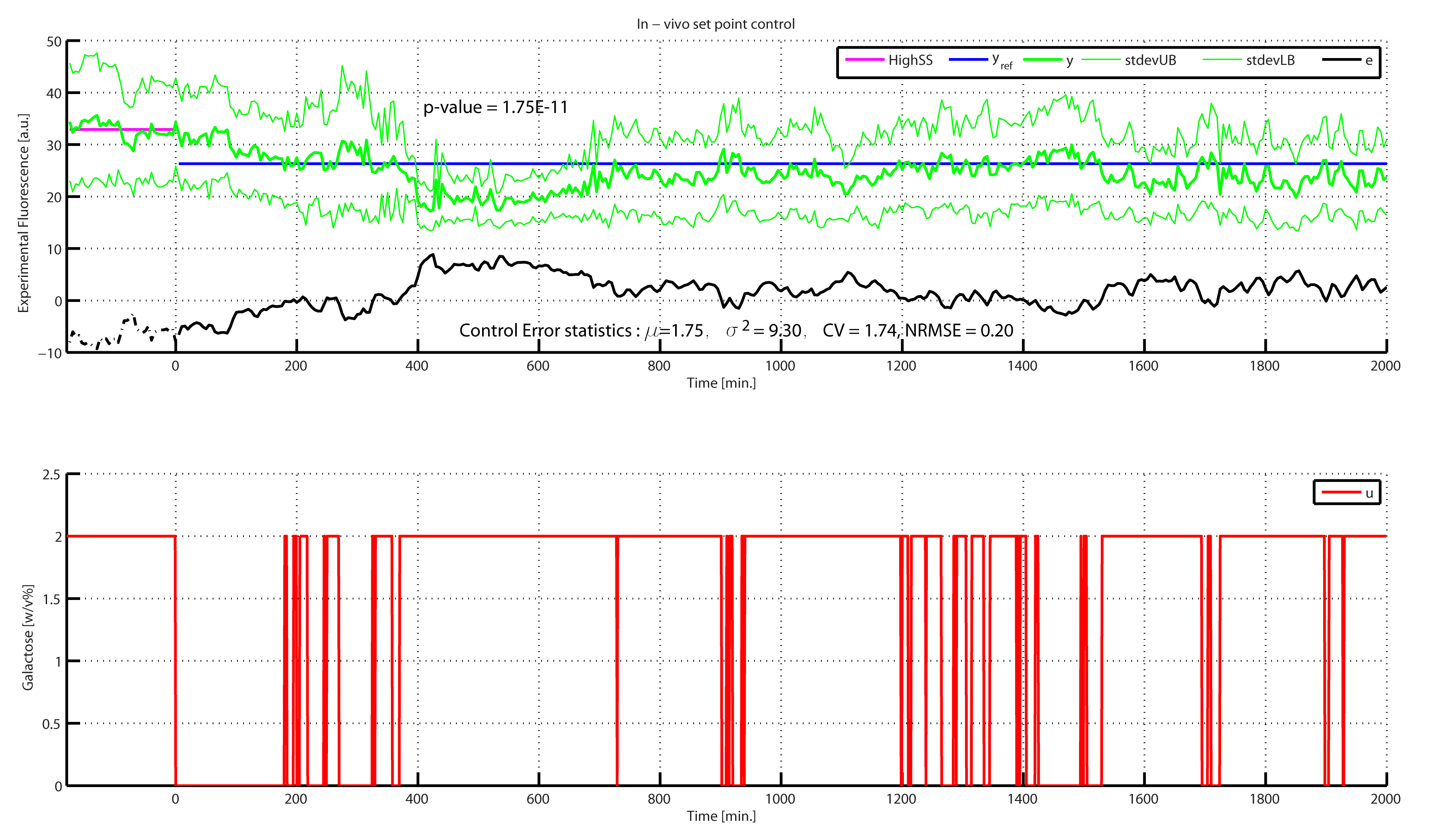

Supplement: Figure S15 — In-vivo set point control experiment for the IRMA network - fluorescence standard deviation. By using the off-line analysis described in the text it is possible to calculate the standard deviation of the fluorescence for each frame acquired during the control. The desired amount of protein ( in blue), the quantified GFP ( green line), the standard deviation's upper and lower bounds (thin green lines) and the control error in black are shown; mean , variance and coefficient of variation of the control error are also shown; the p-value was computed as described in the Supplementary Information text (top panel). The input signal computed by the control algorithm is shown in red (bottom panel). (TIF) [file pcbi.1003625.s015.tif]

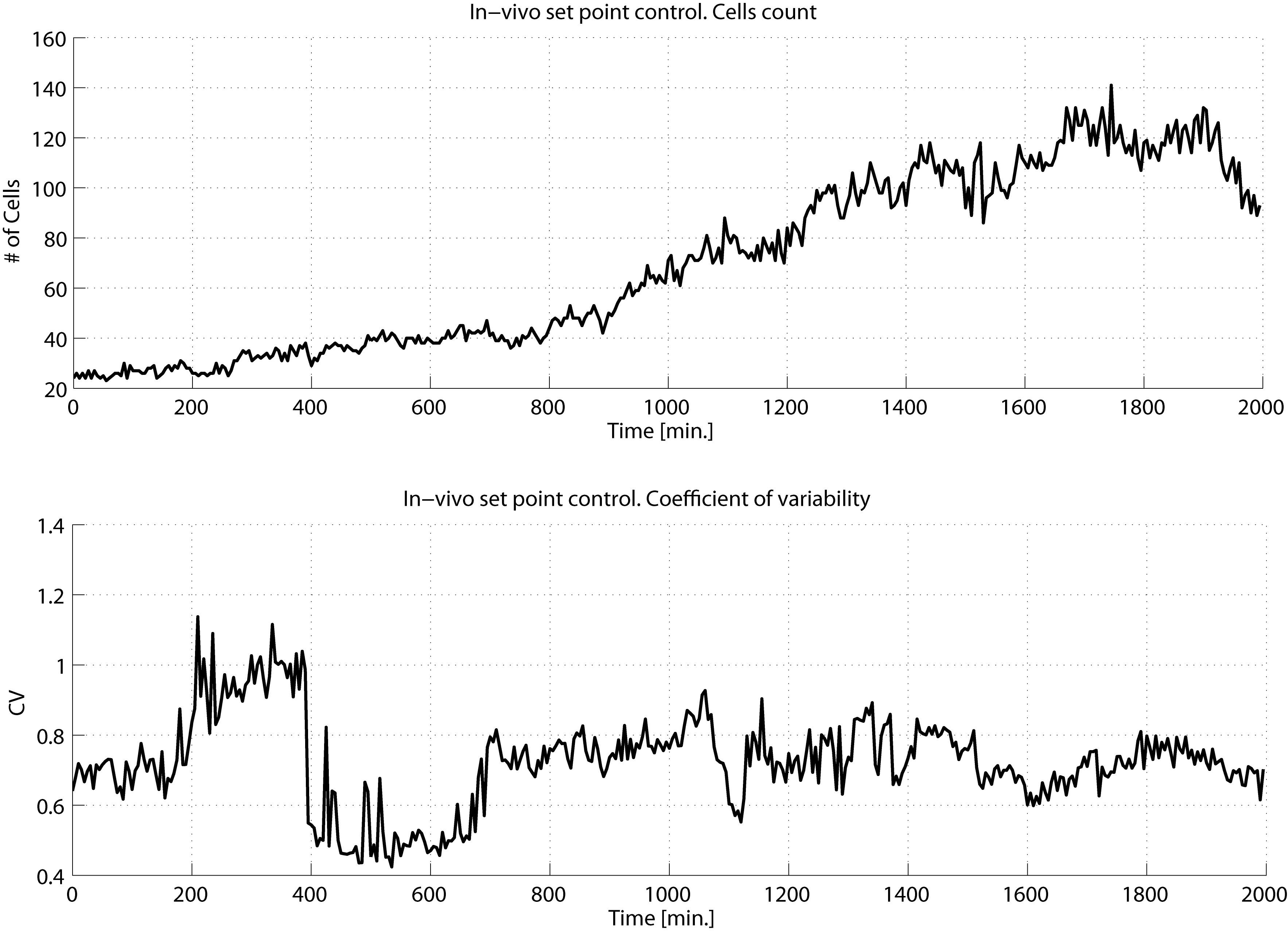

Supplement: Figure S16 — In-vivo signal tracking control experiment for the IRMA network - cell count and coefficient of variation. For the experiment of Supplementary Figure S15, the number of cell (top panel) and the coefficient of variation (bottom panel) are shown. (TIF) [file pcbi.1003625.s016.tif]

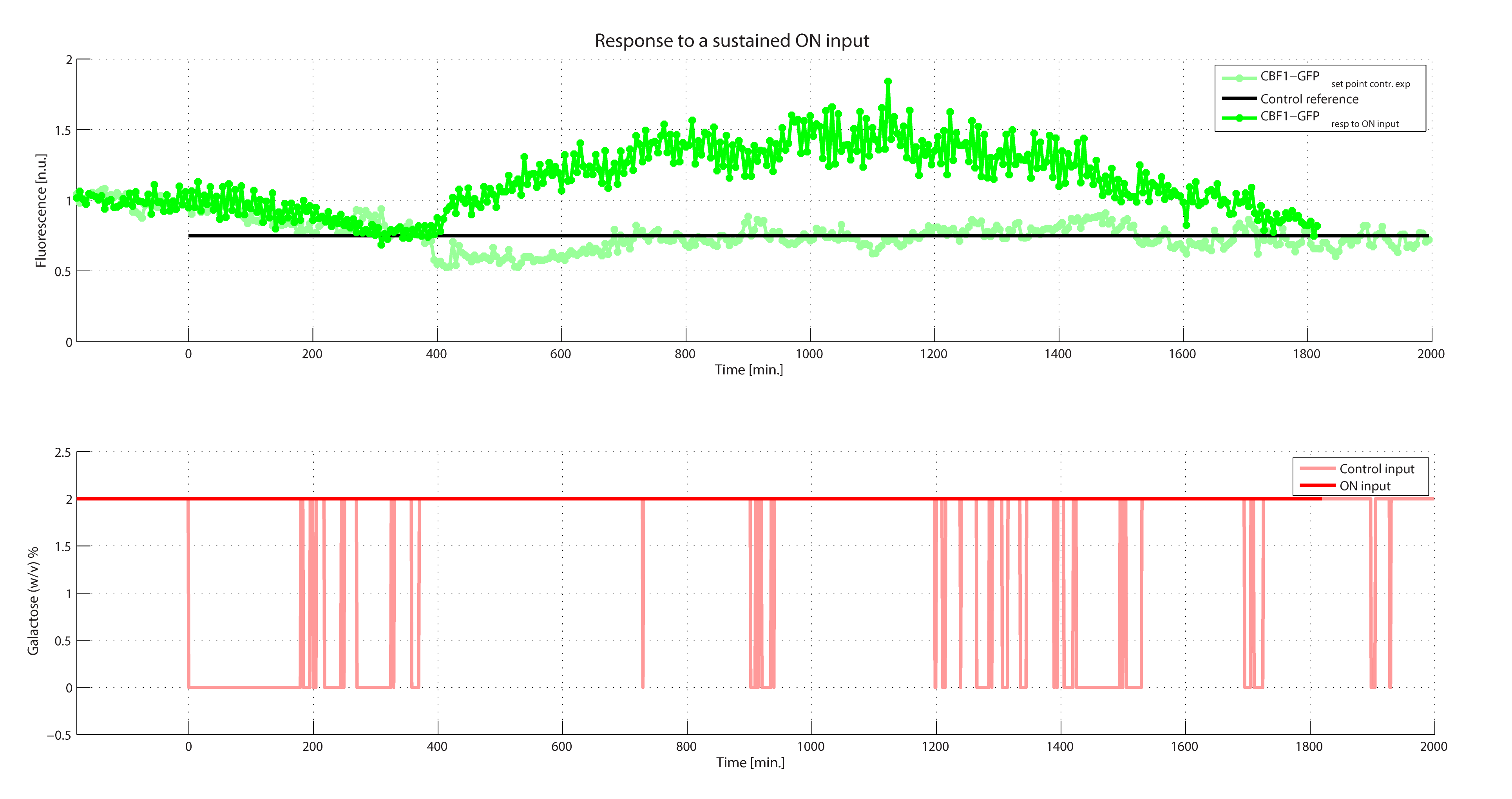

Supplement: Figure S17 — Response to a sustained galactose input for the IRMA network. Green line: fluorescence measured when the cells are treated with galactose for the whole experiment; light green line: fluorescence measured during the in-vivo set point control experiment (Figure 7 - main text); black line: the control reference of the set-point control experiment (Figure 7 - main text); red line: the sustained galactose input provided to the cells population; light red: the input calculated automatically by the control algorithm and used to regulate the production of GFP to the desired level in in-vivo set point control experiment (Figure 7 - main text). (TIF) [file pcbi.1003625.s017.tif]

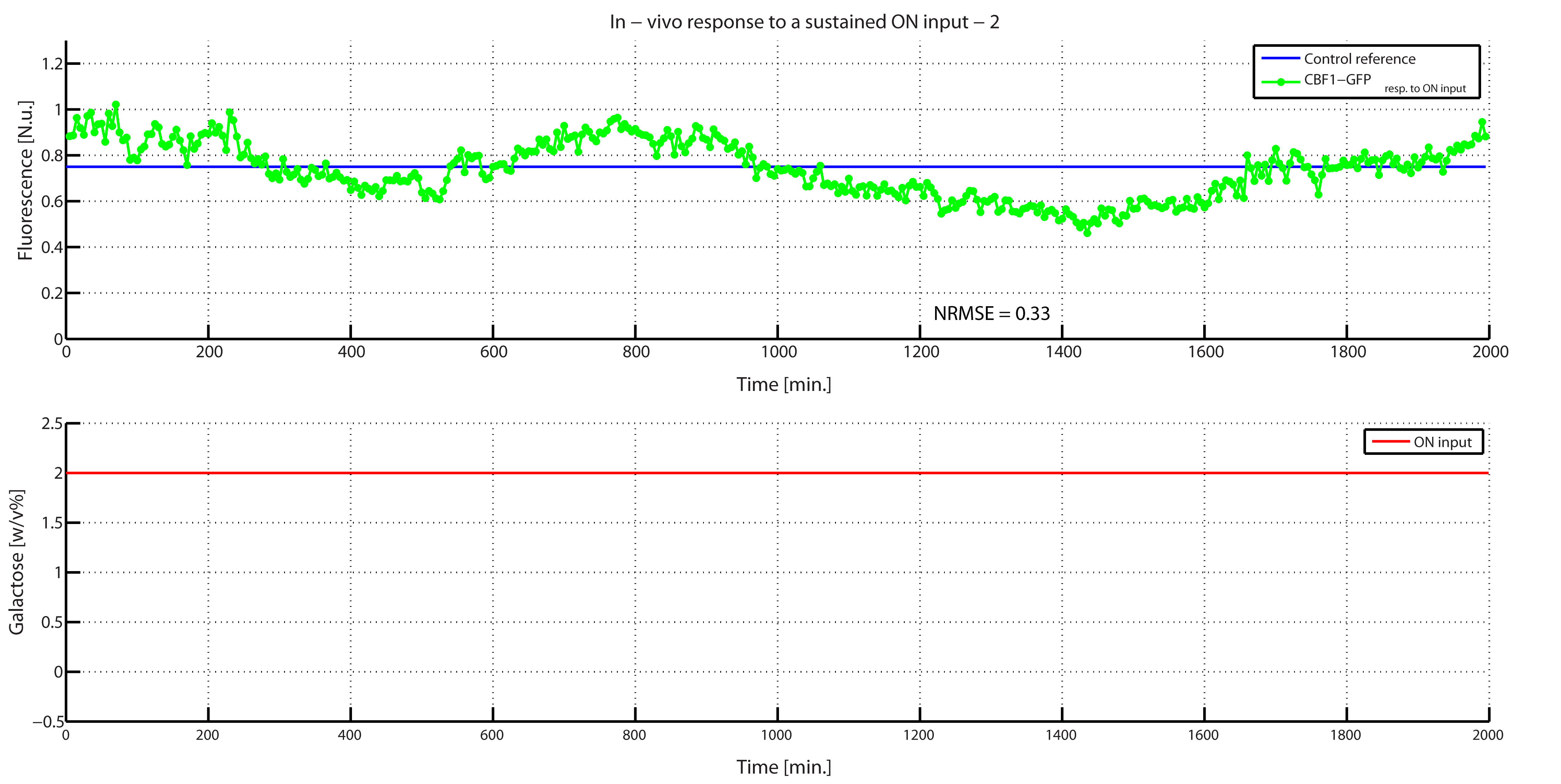

Supplement: Figure S18 — Response to a sustained galactose input for the IRMA network. Green line: fluorescence measured when the cells are treated with galactose for the whole experiment; blue line: the control reference of the set-point control experiment (Figure 7 - main text) (Top panel).(Bottom panel) red line: the sustained galactose input administered to cells. The normalised root mean square error (NRMSE) of the deviation between the blue and the green signal has been reported to be equal to 0.33. (TIF) [file pcbi.1003625.s018.tif]

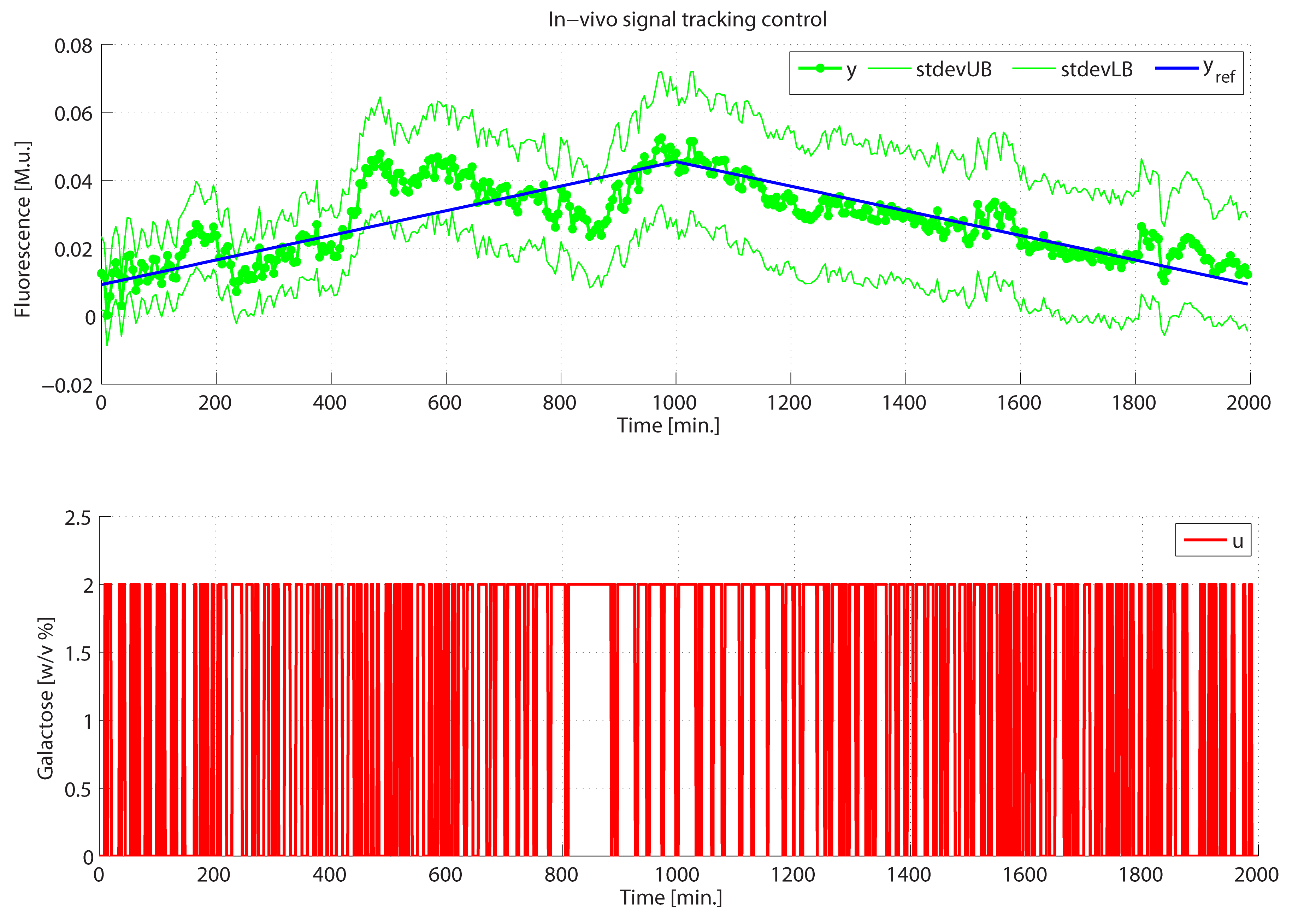

Supplement: Figure S19 — In-vivo signal tracking control experiment for the IRMA network - fluorescence standard deviation. By using the off-line analysis described in the text it is possible to calculate the standard deviation of the fluorescence for each frame acquired during the control. The desired amount of protein ( in blue), the quantified GFP ( green line) and its upper and lower bound of the standard deviation (thin green lines) are plotted (top panel). The input signal computed by the control algorithm is shown in red (bottom panel). (TIF) [file pcbi.1003625.s019.tif]

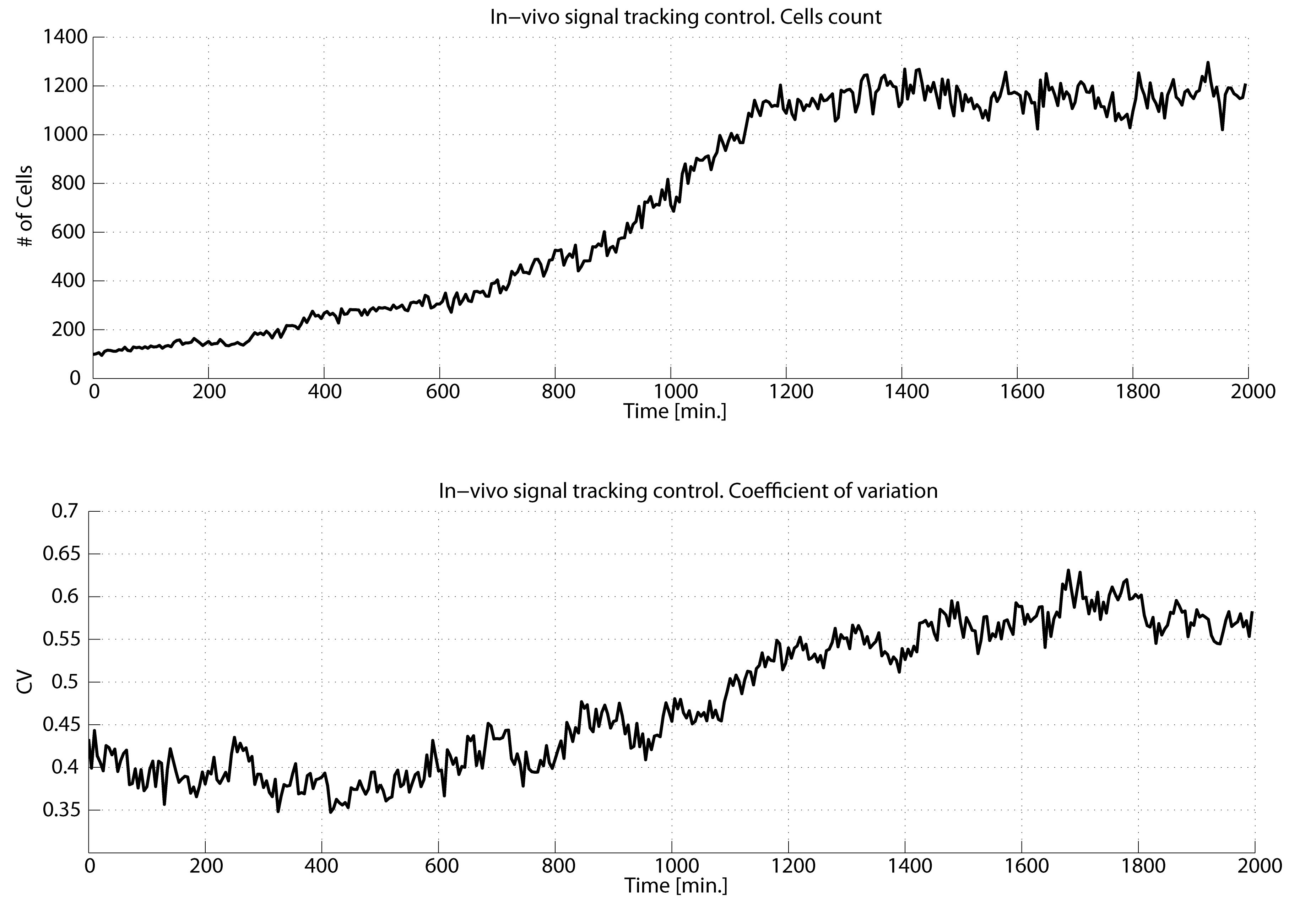

Supplement: Figure S20 — In-vivo signal tracking control experiment for the IRMA network - cell count and coefficient of variation. For the experiment of Figure S19, the number of cell (top panel) and the coefficient of variation (bottom panel) are plotted. (TIF) [file pcbi.1003625.s020.tif]

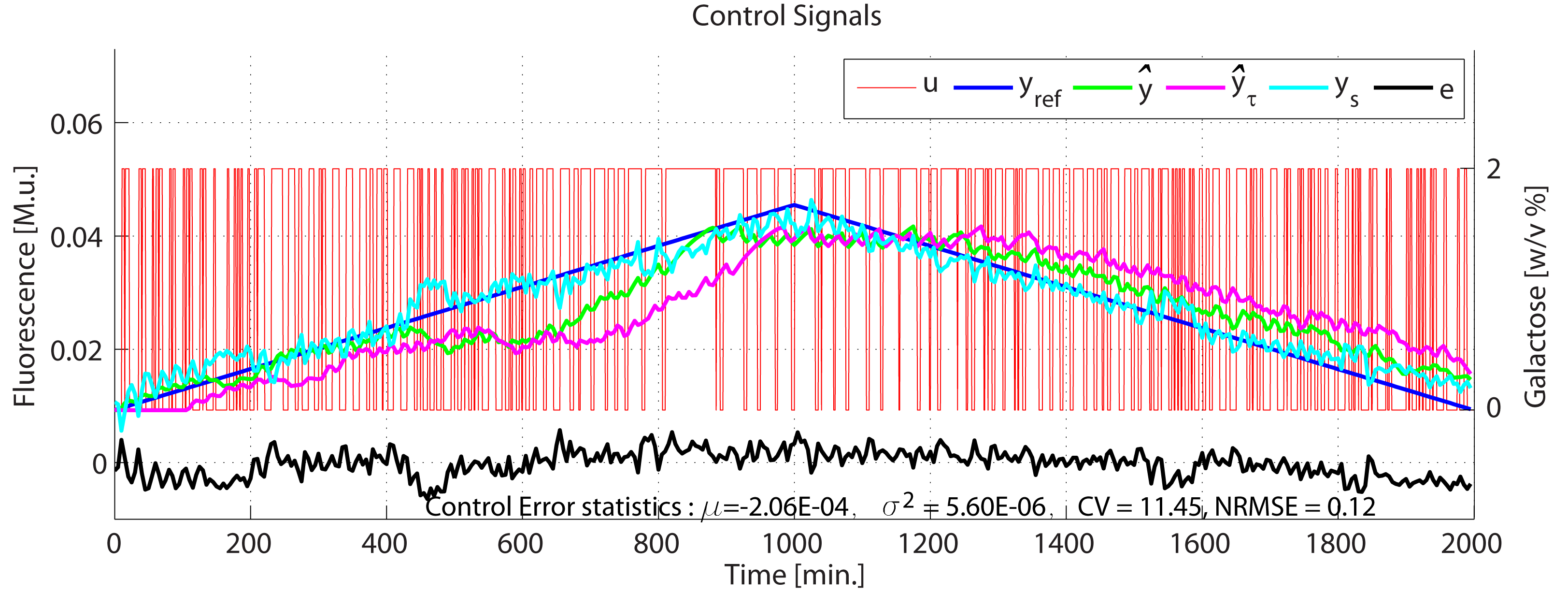

Supplement: Figure S21 — Internal signals of the control experiment in Fig. 8 (main text). Time evolution of the most relevant signals in the control loop are shown. In particular the Galactose concentration in the medium () provided to the cells has been plotted in red, while the output of the delay-free model () and its delayed version () are shown in green and violet respectively. The error signal (black) calculated as the difference between and (cyan) is also depicted; mean , variance and coefficient of variation of the control error are also shown. (TIF) [file pcbi.1003625.s021.tif]

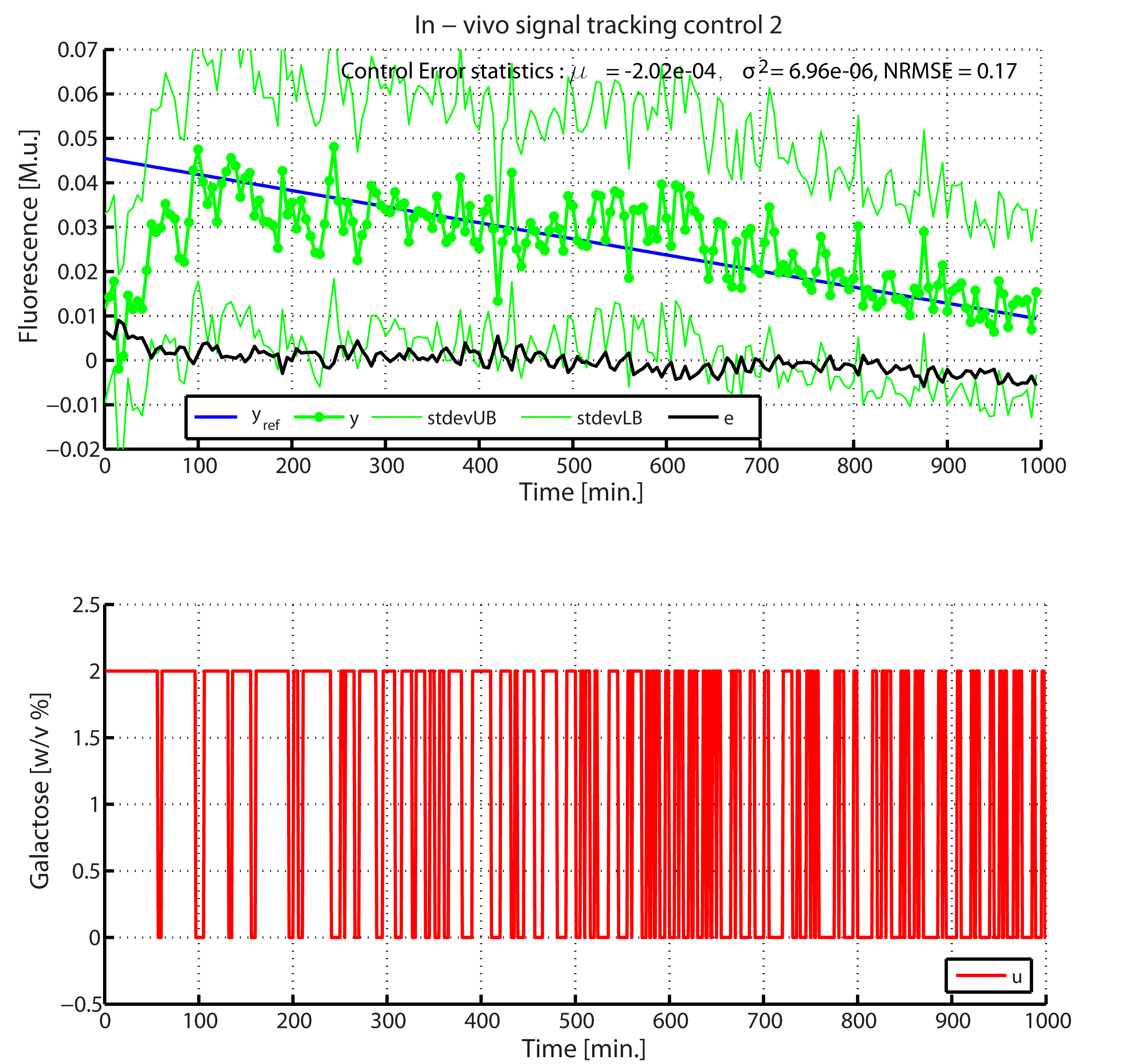

Supplement: Figure S22 — In-vivo signal tracking control experiment 2 for the IRMA network - fluorescence standard deviation. By using the off-line analysis described in the text it is possible to calculate the standard deviation of the fluorescence for each frame acquired during the control. The desired amount of protein ( in blue), the quantified GFP ( green line) and its upper and lower bound of the standard deviation (thin green lines) are plotted; the control error calculated as the difference between the feedback signal and the control reference is shown in black (top panel). The input signal computed by the control algorithm is shown in red (bottom panel). (TIF) [file pcbi.1003625.s022.tif]

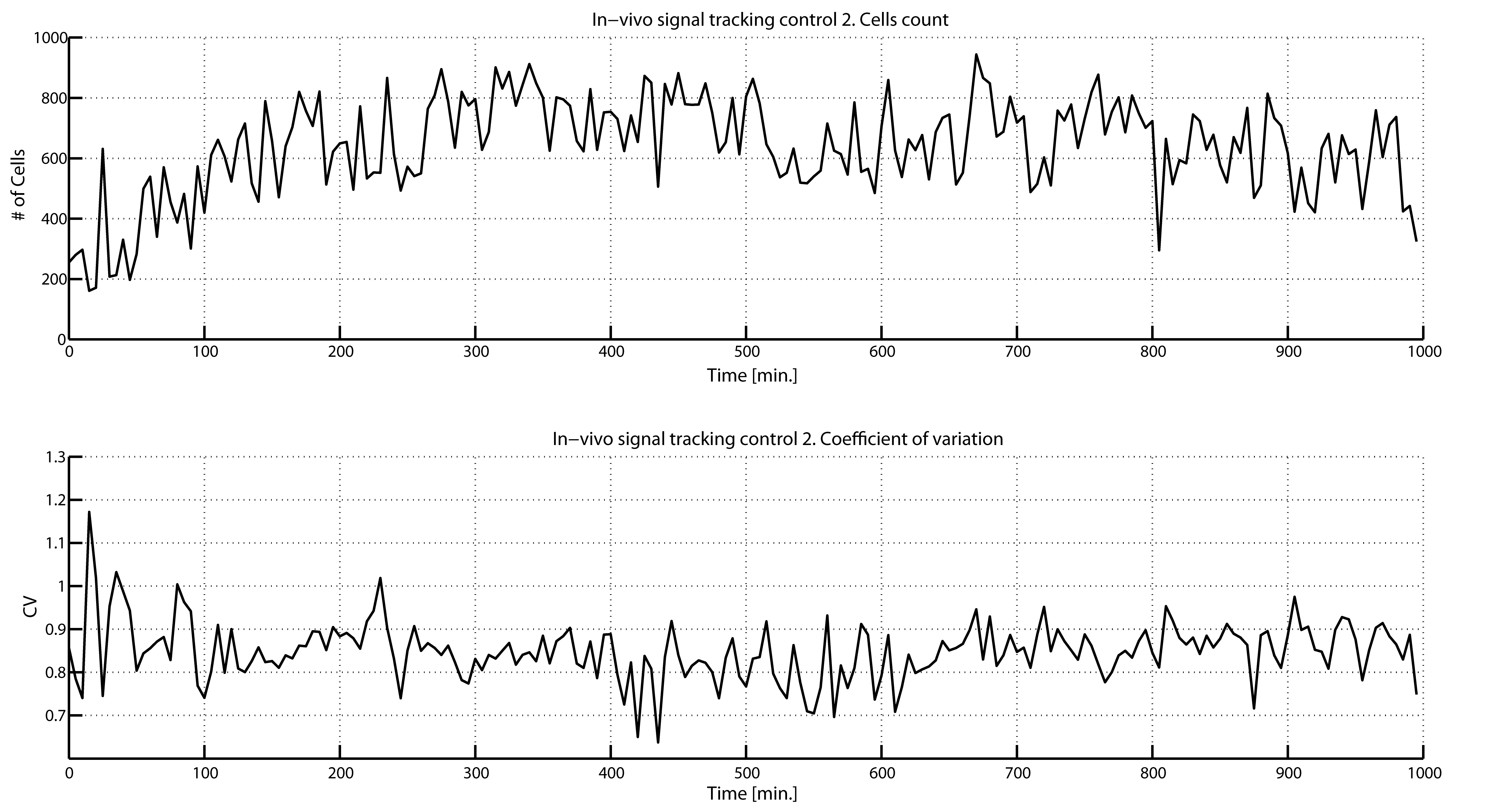

Supplement: Figure S23 — In-vivo signal tracking control experiment 2 for the IRMA network - cell count and coefficient of variation. For the experiment of Figure S22, the number of cell (top panel) and the coefficient of variation(bottom panel) are plotted. (TIF) [file pcbi.1003625.s023.tif]

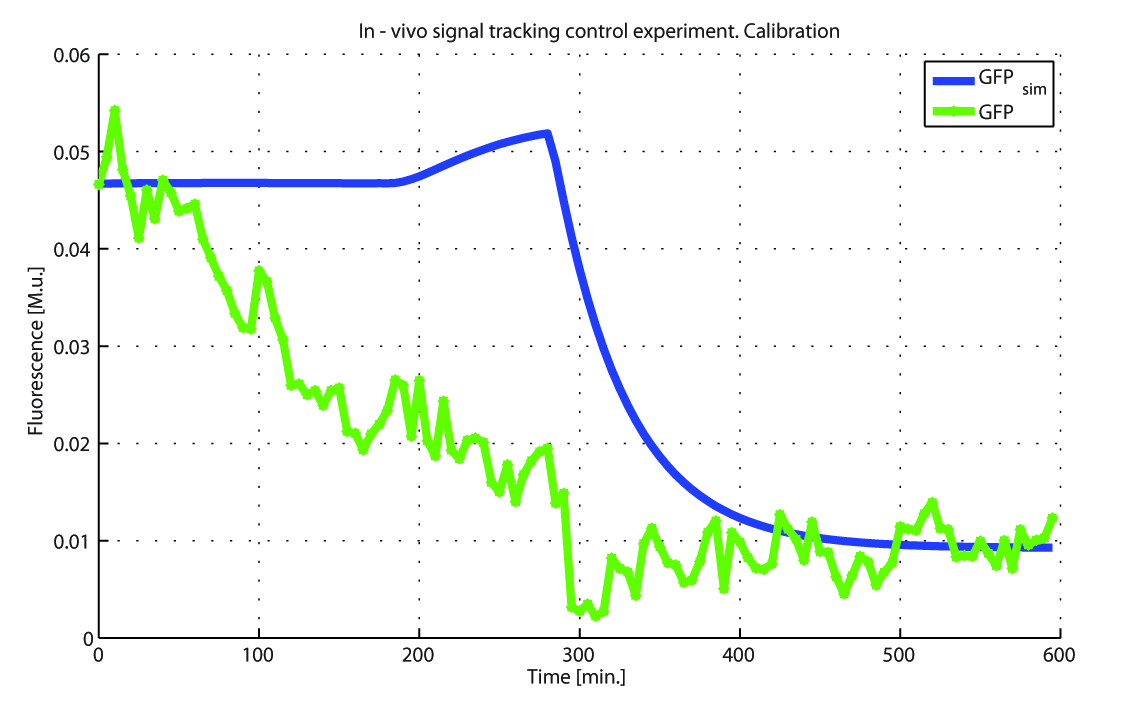

Supplement: Figure S24 — Calibration phase. The calibration data have been reported for the experiment in Fig. 8. The simulated (blue) and quantified (green) Gfp evolution have been used to relate fluorescence data to model predictions (model units). (TIF) [file pcbi.1003625.s024.tif]

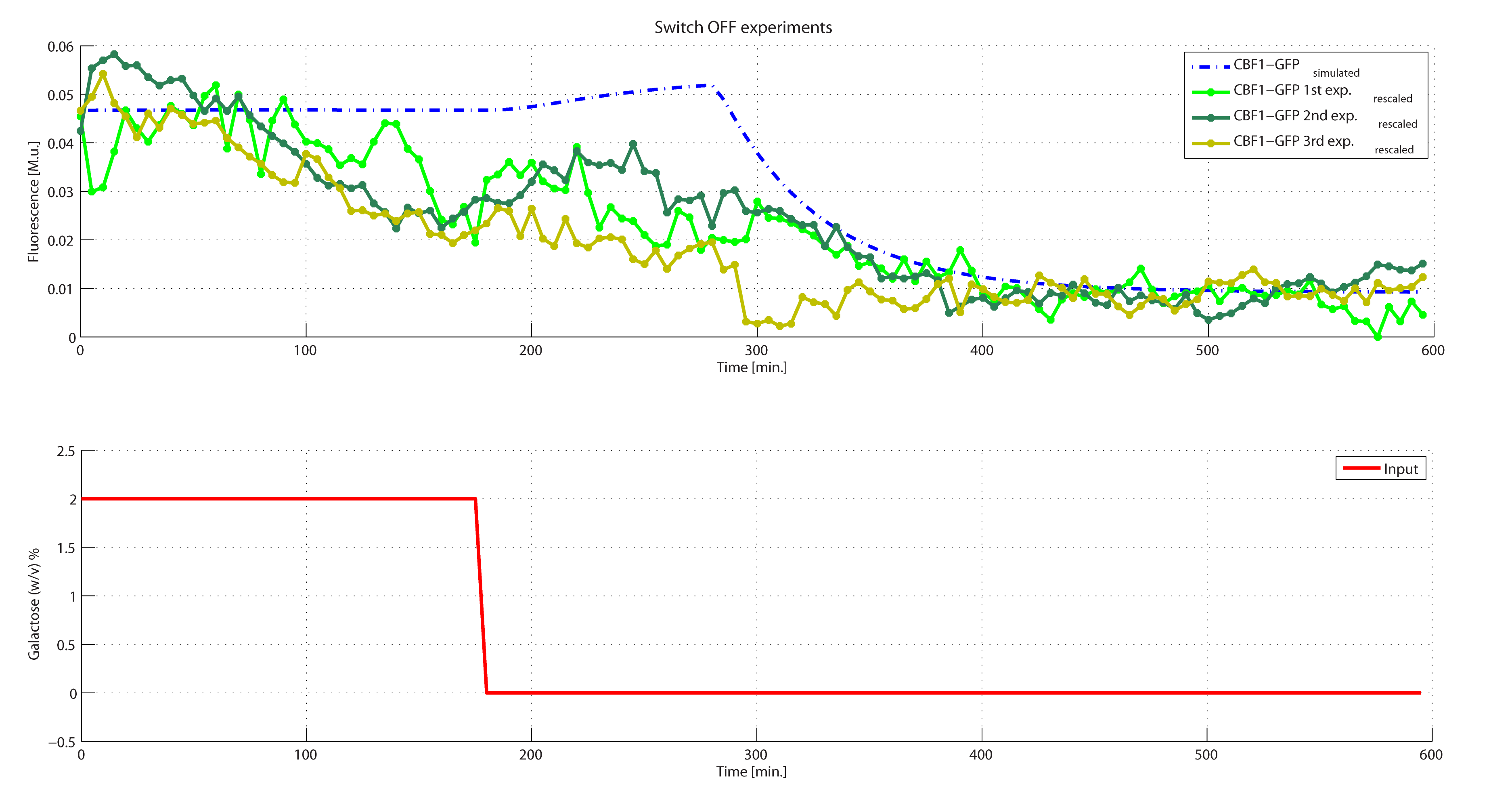

Supplement: Figure S25 — IRMA switch off experiment. Top panel: the green signals represent the measured fluorescence during in-vivo switch - off experiments, the blue signal is the result of in-silico switch off experiment using the dynamical model of IRMA (all the experimental signals are rescaled to the model range). Bottom panel: the input used to perform the experiment; cells have been fed for 180 minutes with galactose (ON signal, 1 for the mathematical model) and for 420 minutes with glucose (OFF signal, 0 for the mathematical model). (TIF) [file pcbi.1003625.s025.tif]
