# Supplementary material for: In-Vivo Real-Time Control of Protein Expression from Endogenous and Synthetic Gene Networks
Source: PLoS Comput Biol. 2014 May 15;10(5):e1003625. doi: 10.1371/journal.pcbi.1003625 (PMC4022480; doi:10.1371/journal.pcbi.1003625)
Supplement: Text S1 — Supplementary information text. All the additional details concerning materials and methods of the present work are here reported. (PDF) [file pcbi.1003625.s026.pdf]

In-vivo real-time control of protein expression from endogenous and synthetic gene networks

- Supplementary Information -

Filippo Menolascina[1,2,†,*], Gianfranco Fiore [1,2,*], Emanuele Orabona [3], Luca De Stefano [3], Mike Ferry [4], Jeff Hasty [4], Mario di Bernardo [2,5], Diego di Bernardo[1,2,‡]
1 TeleThon Institute of Genetics and Medicine (TIGEM), Naples 80131, Italy.
2 Department of Electrical Engineering and Information Technology, University of Naples Federico II, 80125 Italy.
3 Institute of Microelectronics and Microsystems (IMM), CNR, Naples 80131, Italy.
4 Department of Bioengineering, University of California, San Diego, La Jolla, CA 92093-0412, USA.
5 Department of Engineering Mathematics, University of Bristol, UK.
† *Current address*: **Department of Civil and Environmental Engineering, Massachusetts Institute of Technology, Cambridge, MA, USA.**
∗ **These authors contributed equally to this work.**
‡ **E-mail: dibernardo@tigem.it**

Mathematical model of the IRMA synthetic network

IRMA was developed as a testbed synthetic network in yeast for the design and validation of reverse engineering and modelling approaches [1].

IRMA consists of five genes, *CBF1*, *ASH1*, *SWI5*, *GAL4* and *GAL80* and its topology comprises both transcriptional and protein-protein regulation mechanisms ([1]). Figure 2 in the main text shows a schematic diagram of the regulatory interactions among the five genes. The network topology comprises a positive feedback loop from *CBF1* back to itself via *GAL4* and *SWI5*, and a negative feedback loop from *CBF1* back to itself via *ASH1*. A further regulation is present between *SWI5* and *GAL80* via the *GAL10* promoter bound by *GAL4*. Transcription of network genes can be controlled by the presence of Galactose (GAL) in the growth medium, whose presence inhibits transcription of *SWI5* from the *GAL10* promoter.

To capture the dynamics of the network a hybrid model (Supplementary Figure S1) approximating the dynamics in Glucose (F_1) and Galactose (F_2), has been readapted from [1]. Both the vector fields F_1 and F_2 share the same model structure as well as most of the parameters ($\hat{v_3}$, $\hat{k_4}$ and $\hat{\gamma}$ need a specific argumentation) as reported below:

$$\frac{dx_1}{dt} = \alpha_1 + v_1 \left(\frac{x_3^{h_1}(t-\tau)}{(k_1^{h_1} + x_3^{h_1}(t-\tau)) \cdot \left(1 + \frac{{x_5}^{h_2}}{k_2^{h_2}}\right)} \right) - d_1 x_1 \quad (1)$$

$$\frac{dx_2}{dt} = \alpha_2 + v_2 \left(\frac{x_1^{h_3}}{k_3^{h_3} + x_1^{h_3}} \right) - d_2 x_2 \quad (2)$$

$$\frac{dx_3}{dt} = \alpha_3 + \widehat{v}_3 \left(\frac{x_2^{h_4}}{\widehat{k}_4^{\,h_4} + x_2^{h_4}(1 + \frac{x_4^4}{\widehat{\gamma}^4})} \right) - d_3 x_3 \quad (3)$$

$$\frac{dx_4}{dt} = \alpha_4 + v_4 \left(\frac{x_3^{h_5}}{k_5^{h_5} + x_3^{h_5}} \right) - d_4 x_4 \quad (4)$$

$$\frac{dx_5}{dt} = \alpha_5 + v_5 \left(\frac{x_3^{h_6}}{k_6^{h_6} + x_3^{h_6}} \right) - d_5 x_5 \quad (5)$$

where $x_1 = [CBF1GFP], x_2 = [GAL4], x_3 = [SWI5], x_4 = [GAL80], x_5 = [ASH1]$ are the system states. We used Hill functions to model transcription rates from promoters; the multiple regulation on *CBF1* is modelled by the product of two Hill functions (AND regulation). A time delay τ is present in the equation for x_1 modelling the transcription of *CBF1*, which is affected by a 100 minute-long time delay due to the sequential recruitment of chromatin-modifying complexes to the *HO* promoter (which follows binding of *SWI5* and other transcription factors) [2]. A list of all model parameters can be found in Supplementary Table S1 in [1].

Note that the model is hybrid as parameters $\hat{v}_3, \hat{k}_4$ and $\hat{\gamma}$ switch between two different sets of values depending on the carbon source (Galactose or Glucose).

Control algorithm: design and implementation

GAL1 promoter

In this system, the *GAL1* promoter drives the expression of the Gal1-Gfp fusion protein. Thus it can be described as a single input-single output (SISO) dynamical system. The input $u(t)$ describes the presence of galactose or glucose in the growth medium. The output $y(t)$ is the measured average level of fluorescence of the Gal1-Gfp protein in the cell population.

The control objective is a set-point regulation, where the cell population is required to express, over several generations, a fixed amount of fluorescence (control reference $r(t)$). To this end, we designed a simple control algorithm based on a Proportional-Integral regulator (PI) whose output $\hat{u}(t)$ is a function of the control error $e(t)$ (the mismatch between the desired and current output of the system $e(t) = r(t) - y(t)$) defined as:

$$\hat{u}(t) = K_p \cdot e(t) + K_I \cdot \int_0^t e(t)dt \quad (6)$$

Since cells can consume either galactose or glucose in a mutually exclusive manner, the continuous signal $\hat{u}(t)$ has to be decoded into a discrete signal denoting either galactose ($u(t) = 2$) or glucose ($u(t) = 0$).

The above constraint allows an analogy with the problems faced in the design of feedback control strategies for power electronic circuits [3]. Here, switches and SCRs (silicon controlled rectifiers) can

only be turned on or off, some output is typically measured or estimated and, particularly in industrial applications, compensating noise and external disturbances is of utmost importance.

The simplest and most widely used control technique in this context is to use the PI regulator coupled to a PWM (Pulse Width Modulation) control strategy with a classic limited integral anti-windup [4]. This is also the strategy we adopted to control the cell population. In the simplest feedback implementation of the PI-PWM, a sawtooth signal is compared with $\hat{u}(t)$ in order to modulate the width of a rectangular pulse train, which is then used as control input (see [5] for further details). Namely, let

$$\eta(t) = \alpha + \beta(t \bmod T) \tag{7}$$

be the sawtooth signal; then

$$u(t) = \begin{cases} 0, & \text{if } \eta(t) - \hat{u}(t) > 0, \\ 2, & \text{otherwise} \end{cases}$$

To control the *GAL1* promoter we chose the sawtooth wave parameters as follows: $\alpha = 0$, $\beta = 2$ and $T = 5min$. The gains of the PI controller, namely $K_p = 6$ and $K_i = 0.3$, were tuned following the Cohen-Coon strategy ([6]) using a linear transfer function describing the *GAL1* promoter previously derived in [7]:

$$G_{approx}(s) = \mu \frac{e^{-ds}}{1 + \Theta s} \tag{8}$$

with parameters $\mu = 0.28$, $d = 68$ and $\Theta = 134$.

IRMA network

IRMA can be modeled as an input-output system where the input u models the presence/absence of Galactose and the output y is the concentration of one of its genes, namely *Cbf1* (x_1). Note that the input acts nonlinearly on the dynamics of the network as the presence of Galactose changes the values of all the Galactose-dependent parameters (namely $\hat{v}_3, \hat{k}_4$ and $\hat{\gamma}$). Moreover, as soon as Glucose is administered to the cells, these stop responding to Galactose, even if it is still present in the medium. Therefore, the control input (interpreted as Galactose concentration of 2 w/v% in the total volume of fluid reaching yeasts) is restricted to be either ON ($u = 1$) or OFF ($u = 0$). The system output $y = x_1$ cannot be measured directly as a concentration. Instead, the cells were engineered so that CBF1p is fused with a GFP, the green fluorescent protein [1]. In this way, higher concentrations of Cbf1p are associated to higher levels of fluorescence. Moreover, as outlined in [8], the amount of estimated fluorescence can be directly related to the actual number of molecules. From a control perspective, the gene network model is, therefore, a highly nonlinear, hybrid, time-delayed dynamical system of the form:

$$\dot{x} = \begin{cases} F_1(x, x(t-\tau), \mu), & \text{if } u = 0, \\ F_2(x, x(t-\tau), \hat{\mu}), & \text{if } u = 1 \end{cases}$$

where $x = [x_1\ x_2\ x_3\ x_4\ x_5]^T$, μ is the vector of parameter values in Glucose ($u = 0$) and $\hat{\mu}$ is the vector of parameter values in Galactose ($u = 1$).

Hybrid systems are often used to model gene networks (e.g. see [9–11]), where it is quite common to observe threshold dependent and switch-like activation or inhibition functions governing the dynamics of protein-protein or protein-gene interactions.

From a control viewpoint, the task is to regulate the expression of *CBF1* to some desired value, say $r(t)$ by modulating the control input $u(t)$ (the carbon source being administered to the cell). In what follows, we will ignore phenomena like Zeno behaviors (which would imply the gene network to switch on and off an unbounded number times in a finite and bounded length of time). To solve this problem, the challenge is to design an effective, yet simple, control strategy able to cope with the many unavoidable

constraints, which characterize biological systems (i.e. high levels of noise, incomplete knowledge and unmodelled dynamics). Cells are living organisms and they resist external actions very effectively.

Moreover, it is important to minimize the need of knowing in great detail the biological process to be controlled, in order to make the control strategy as general as possible and applicable to any process of interest.

The main design constraint that needs to be taken into account when synthesizing a controller for a biological system such as IRMA is that the cells tend to consume Glucose as their primary carbon source, even when galactose is present, since they obtain energy from it at a lesser energetical cost [12]. Moreover GLU-genes repress GAL genes as outlined in [13]; therefore our control input is binary: either galactose or glucose, but not both.

As in the case of the *GAL1* promoter, we used the PI-PWM control strategy for the set-point control task. The sawtooth wave parameters for the PWM were set to $\alpha = 0$, $\beta = 10E - 5$ and $T = 5\ min$. In this way, the amplitude of the signal is 10% of the *Cbf1* level at steady state in Glucose and the period is twice the settling time of *Cbf1*. With this choice, the constraint is satisfied of the control input being binary.

In the case of the signal tracking control task, in order to compensate for the estimated delay of 100min in the *CBF1* gene transcription, a strategy was implemented, which is inspired by the classical Smith Predictor scheme in [14], as shown in Supplementary Figure S2. The delay-free version of IRMA's model (block M in Supplementary Figure S2) is used to produce a numerical estimate of the anticipated system output $\hat{y}$. This is then delayed and subtracted from the quantified fluorescence output, y, of the network so as to produce a signal which is filtered via a linear filter F and added back to y. The filter was designed empirically to suppress high frequency noise entering the control loop and is characterized y:

$$F(s) = \frac{1}{1 + 20s} \tag{9}$$

The output y_s of the delay compensation scheme is then compared to the desired output (y_{ref}) to obtain the error signal e. A Proportional-Integral controller takes e in input and computes the control signal $\hat{u}(t)$ with the gains $K_p = 175.6$ and $K_I = 2.11$. These gains were found with the Cohen-Coon method, as previously described for the *Gal1* promoter; in the case of IRMA the parameters of the transfer function in eq. (8) were found to be $\mu = 0.0467$, $d = 146.85$ and $\Theta = 667.62$. A comparison between the step response of the linear approximation with the non linear model is shown in Supplementary Figure S3. The computed analog control signal $\hat{u}$ is then converted into binary values by means of the PWM modulator described above that feeds both the physical plant and the model used for delay compensation with the same binary input u, thus closing the feedback loop.

Control algorithm implementation

The control strategy described in the previous section was implemented as a Finite State Automaton in MATLAB (Mathworks Inc.) for the set-point regulation of both *GAL1* promoter and the IRMA network.

The Finite State Automaton implementing the PI-PWM control algorithm is very simple: at each step (k) an image is acquired by the microscope, and the normalized fluorescence signal is computed thanks to an image segmentation algorithm described in the next section. The fluorescence signal $y_s(k)$ is compared against the reference signal $y_{ref}(k)$, to obtain the error $e(k)$. The control input $u(k)$ is then computed using the discrete-time implementation of the PI controller discussed in [4].The control input $u(k)$ is used to determine the duration of the pulse of Glucose or Galactose by means of the PWM strategy. The duration of each pulse corresponds to the time interval during which the syringe loaded with Galactose remains higher than the one containing Glucose (or vice-versa). At the next instant $(k+1)$ a new image is acquired and the feedback computation takes place. The error $e(k+1)$ is available for a new control iteration and each step is repeated again.

In the case of the signal tracking control task for the IRMA network, the Finite State Automaton implementing the control algorithm is more complex, since in this case there is the need of simulating the IRMA model M in the Predictor block, necessary for the delay compensation. As represented in Supplementary Figure S4, at the k-th step, $e(k)$ is known, thus it is possible to calculate the control input $u(k)$ to be applied to both the plant and the IRMA model M via the PI controller [4]. The control input is then used to compute the duration of the pulse of Glucose or Galactose by means of the PWM strategy.

In-silico analysis of the control algorithm for the IRMA network

Given the complexity of the IRMA model, prior to the experimental implementation of the control strategy *in - vivo*, we decided to test and validate the performance of the control scheme *in - silico*.

In the numerical implementation, several alternative design options were evaluated. For example, it was found that using a nonlinear model for delay compensation (in the block labelled as 'M' in Supplementary Figure S2) gives a comparable performance to that obtained when using a linearized one (as suggested in [14]).

In addition, we performed numerical simulations of the control scheme against uncertainties in the delay estimation by considering the scenario where the delay τ is set to zero. The results are presented in Supplementary Figure S5 and Supplementary Figure S6 showing the ability of the control scheme to guarantee a reasonable performance also in this case.

Finally, we explored the effects of simplifying the control strategy further by removing the prediction scheme used to compensate the delay. Although a simpler control strategy may decrease the overall performance, the reason for taking this step is that we prefer to sacrifice precision in favour of a control strategy which can easily be used to control other gene networks and proteins, without requiring a dynamical model of the system to be controlled.

In-silico results reported in Supplementary Figure S7 and Supplementary Figure S8 show the performance of such a simple PI/PWM control strategy when implemented to control gene expression levels in IRMA. The in silico experiments confirm that this strategy force effectively the *Cbf1* concentration to track the desired reference signal when the delay $\tau = 0$.

Microscopy and image analysis

The closed-loop control platform described above, employs an inverted epifluorescence Nikon-TI Eclipse microscope. The microscope is programmed to acquire two types of images: *(a)* a phase contrast image (phase-contrast) and *(b)* two fluorescence images (one for the green spectrum for GFP and one in the red spectrum for Sulforhodamine B). The red dye Sulforhodamine B is added to the galactose medium and it is used to check for the proper administration of the control input. Once cells have been imaged, image analysis methods can be applied to estimate their fluorescence [15]. To this end, we developed a custom image processing algorithm to fully exploit the peculiarities of our platform. [16]

Our aim was to carry out both cell segmentation and cell-tracking, maximizing sensitivity as primary objective, and then refining the results by improving specificity. We based our segmentation method on global edge linking system. Yeast cells in phase contrast images occur in clustered, low intensity, convex and often quasi-circular shapes surrounded by a white halo (Supplementary Figure S9 C).

The contrast between the pixels belonging to the cells and the pixels belonging to the halos is usually so high that edge points can be detected by the evaluation of the magnitude of the gradient calculated in each point of the image (Supplementary Figure S9).

Due to the shape of yeast cell, edge points can be connected with the Circular Hough Transform (CHT) [17]. CHT can detect almost all cells within the image, even when cells edges overlap; unfortunately

the CHT algorithm is computationally expensive (both in memory and time) and shows limited specificity capabilities.

Therefore, in order to limit the computational cost associated to the algorithm, we introduce a pre-processing stage of the image to select the regions in the image where cells are located (Supplementary Figure S9 B). Such regions are identified via a mask obtained through morphological operations (aperture, closure and filling) and thresholding (see Supplementary Figure S9 A). False detections are reduced as a result of the segmentation of these regions. Moreover, since the area of the regions in the image containing cells is smaller than the area of the background, computational time is considerably reduced.

Once cells have been located in the phase contrast image, a binary filter is used to detect only pixels of the GFP fluorescence field within cells. Let us define the fluorescence field image I as:

$$I : (p) \in \Omega \subset \mathbf{N}^2 \tag{10}$$

then

$$I(x, y) \in \left[0, 2^{-L} - 1\right] \subset \mathbf{N} \tag{11}$$

with x and y generic coordinates and L the number of bits used for image encoding and ω the set of intensity values the pixel in the image can assume.

The mask image $\hat{M}$ can be similarly defined as:

$$\hat{M} : (p) \in 0, 1 \tag{12}$$

where $\hat{M}(x, y) = 1$ denotes a cell belonging pixel while $\hat{M}(x, y) = 0$ indicates background pixels.

The latter class of pixels is useful to estimate the amplitude of the background signal, which can be subtracted the raw signal to obtain a normalised fluorescence intensity. In order to compute the normalised signal, we use the following equation:

$$\mathrm{GFP}_{\mathrm{avg}} = \underbrace{\sum_i \sum_j I(x, y) \cdot \hat{M}(x, y)}_{\text{raw GFP signal}} - \underbrace{\sum_i \sum_j I(x, y) \cdot (1 - \hat{M}(x, y))}_{\text{background signal}} \tag{13}$$

with i and j spanning the rows and columns, respectively, of the arrays. $\neg\hat{M}(x, y)$ is a transformation of $\hat{M}$ that is simply meant to complement the binary values of the original matrix (so as to select image areas not belonging to cells).

The quantity GFP_{avg} is the quantified fluorescence output y used by the control algorithm to define the control input to the cells.

In order to evaluate single cell fluorescence for each frame acquired during the experiment, an off-line analysis (not during the control) is performed by using the same principles of the algorithm described above; a mask $\hat{M}$ is built for each cell in a frame, it allows to calculate the fluorescence of the selected cell only (also the background value is subtracted). This analysis is useful to compute fluorescence histograms for each frame (see Supplementary Movie 1 and 2) and to calculate the standard deviation and the coefficient of variability of the output for each *in - vivo* experiments performed (see Supplementary Figures S10, S11, S12, S13 and S14 -S15-S16-S19-S20-S22-S23).

Quantitative analysis of the experimental results for the IRMA network

The experimental results presented in the main text can be further analyzed by considering the internal signals involved in the computation of the control action. Supplementary Figure S21 presents these data for the experiment presented in Fig. 8 (main text). The close matching between the output signal y_s

and the desired output behaviour y_{ref} is reflected in the magnitude and shape of the error signal e. It is interesting to observe that, in the first 100 minutes, the system is reacting without any delay to the frequently switching input provided to it. We argue that this phenomenon can be explained by considering the molecular mechanisms giving rise to the time delay τ: as outlined in [2], the delay is due to the chromatin remodelling step needed to initiate the transcription under the control of the HO promoter. Therefore, we hypothesise that fast switching between Galactose and Glucose attenuates the effect of the time delay by inhibiting a complete remodeling of the chromatin.

For the *in-vivo* signal tracking control experiment reported in Figure 8 of the main text and in Supplementary Figure S19, we computed the control error as the difference between the average fluorescence of the cell population (output) and the control reference, and reported its mean μ, variance σ, and, coefficient of variation $CV = \frac{\mu}{\sigma}$ in Supplementary Figure S21. It can be appreciated that the control error is much smaller than the fluorescence signal, demonstrating that the control action is able to keep the signal close to the reference.

As concerns the set point regulation experiment reported in Figure 7 of the main text and in Supplementary Figure S15, we report the control error, computed as described before, and shown in Supplementary Figure S15. In this case, we also performed an additional statistical analysis to test the control action performance in regulating the protein expression level to the desired set-point. Indeed, due to cell-to-cell variability, the fluorescence level in the cell population varies among the cells. Referring to Supplementary Figure S15, we considered two classes of events: (NC) the fluorescence measured in single cells during the first 180 minutes of experiment, when No Control input is applied; (C) the fluorescence measured in single cells after the first 180 minutes of experiment, when the Control action has began. We then compared the control error in class (NC) (dashed black line in Supplementary Figure S15) to the control error in class (C) (solid black line in Supplementary Figure S15) using a one-tail t-test to check if we can reject the null hypothesis $H_0 = e_{NC} \leq e_C$, where $e_.$ represents the control error. We obtained a significant $p - value$ of 1.75E-11, that demonstrates that despite the cell-to-cell variability (see standard deviation bounds in Supplementary Figure S15) the control action is really effective.

Microfluidic device fabrication protocol

Replica molding technique has been used to obtain replicas of the device presented in [18] by Ferry and colleagues. Before the fabrication of the microfluidic devices the master is exposed to chlorotrimethylsilane (Sigma-Aldrich Co.) vapours for 10 min so as to create an anti-sticking silane layer for PDMS. A 10:1 mixture of PDMS prepolymer and curing agent (Sylgard 184, Dow Corning) is prepared and degassed under vacuum for 1 hour. Then the mixture is poured on the patterned, and to facilitate the polymerization and the cross-linking, it is cured on a hot plate at 75°C for 3h. After this step the PDMS layer, containing the microfluidic channels, is peeled from the master and it is cut with a scalpel to separate the single devices; holes are bored through them with a 20-gauge blunt needle in order to create fluidic ports for the access of cells and liquid substances. The PDMS layers obtained are rinsed in isopropyl alcohol in a sonic bath for 10 min to remove debris. For each PDMS piece containing microchannels a thin glass slide (150um) is cleaned in acetone and isopropyl alcohol in a sonic bath for 10 min for each step. Finally the PDMS layers and glass slides are exposed to oxygen plasma in a RIE (Reactive Ion Etching) machine for 10 s and brought into contact forms a strong irreversible bond between two surfaces. As last step all devices were checked for faults inside and outside the channels.

Experimental setup

The experimental setup is the same for both strains of cells used in this study. On day 0 batch cultures are inoculated in 10 mL GAL/RAF+Sulforhodamine B (Sigma-Aldrich) (2%) Synthetic Complete medium (SC). On day 1 the batch culture is diluted at intervals of 12 hours (final OD_{600} 0.01). On day 2, 60mL syringes (Becton, Dickinson and Company, NJ) filled with 10 mL SC+GAL/RAF (2%) and SC+GLC

(2%) media are prepared, as well as sink syringes (filled with 10 mL ddH2O); capillaries and needles are used to allow connection to the microfluidic device. Temperature in the microenvironment sorrounding the moving stage of the microscope is allowed to settle at 30 °C. Before connecting media and sink syringes, the microfluidic device MFD0005a wetting is carried out as described in [18]. After air bubbles are removed, media and water filled 60 mL syringes are attached to the device and correct functioning is checked by inspecting the red-fluorescence emitted by Sulforhodamine B as a result of the automatic height control of syringes. This allowed us to carry out a correct calibration of the actuation strategy before the actual experiment is run. At this point cells (IC18 or yGIL337 strain) are injected in the microfluidic device by pouring the batch culture in a 60 mL syringe similar to the ones used to media and sinks. Once cells are trapped in the defined area (see [18] for details) Perfect Focus System is activated to assist autofocusing during the experiment and the microscope is programmed to acquire images at every 5 minutes interval: phase contrast (40 ms, exposure time) and epifluorescence images (green fluorescence, 300 ms exposure time; red fluorescence, 100 ms exposure time) were acquired to allow the control algorithm to (a) locate the cells (phase contrast images) (b) quantify the synthesised GFP (green fluorescence) and (c) verify the correct administration of Galactose/Glucose (red fluorescence).

Each signal tracking experiment starts with an initial calibration phase, needed to establish a linear relationship between the fluorescence units obtained a read out of the cellular state and the arbitrary units the mathematical model is based on. In order to obtain this conversion, during the first 600 min we supply SC+Galactose/Raffinose for 180 min and Glucose for 420 min. This input signal is meant to (a) limit the stress on cells that were mechanically loaded and (b) obtain the whole fluorescence dynamical range spanning the lower and upper steady states. Imaging is carried out using a Nikon TI-Eclipse microscope equipped with a 40X objective. Fluorescence images are taken using FITC (excitation 460/40 nm, emission $510nm/$ 50nm) and TRITC (excitation 530/30 nm, emission $590nm/$ 60nm) filters. Images are acquired by a Peltier-cooled Andor Clara camera controlled by Nikon Instrument Software v.3.10.

A set point experiment differs from the previously described since the control scheme adopted (PI - PWM cascade without prediction block) is not based on the mathematical model of the synthetic gene network; thus the initial calibration phase is not required. To calculate the maximum amount of protein that the cell population can express, at the beginning of the experiment, the cells are fed with SC+Galactose/Raffinose for 180 min thus, the set point is calculated as a percentage of the average of the fluorescence values measured during these first minutes; then the control starts (it lasts for 2000 min).

References

1. Cantone I, Marucci L, Iorio F, Ricci MA, Belcastro V, et al. (2009) A yeast synthetic network for in vivo assessment of reverse-engineering and modeling approaches. Cell 137: 172.

2. Cosma MP, Tanaka T, Nasmyth K (1999) Ordered recruitment of transcription and chromatin remodeling factors to a cell cycle–and developmentally regulated promoter. Cell 97: 299–311.

3. Kassakian JG, Schlecht MF, Verghese GC (1991) Principles of power electronics, volume 46. Addison-Wesley Reading, USA.

4. Teel AR, Moreau L, Nešić D (2004) Input to state set stability for pulse width modulated control systems with disturbances. Systems & control letters 51: 23–32.

5. Banerjee S, Verghese GC (2001) Nonlinear phenomena in power electronics. IEEE press New York.

6. Aström KJ, Murray RM (2010) Feedback systems: an introduction for scientists and engineers. Princeton university press.

7. Fiore G, Menolascina F, di Bernardo M, di Bernardo D (2013) An experimental approach to identify dynamical models of transcriptional regulation in living cells. Chaos: An Interdisciplinary Journal of Nonlinear Science 23: 025106–025106.

8. Gordon A, Colman-Lerner A, Chin TE, Benjamin KR, Yu RC, et al. (2007) Single-cell quantification of molecules and rates using open-source microscope-based cytometry. Nature methods 4: 175–181.

9. De Jong H (2002) Modeling and simulation of genetic regulatory systems: a literature review. Journal of computational biology 9: 67–103.

10. Gouzé JL, Sari T (2002) A class of piecewise linear differential equations arising in biological models. Dynamical systems 17: 299–316.

11. Ahmad J, Bernot G, Comet JP, Lime D, Roux O (2007) Hybrid modelling and dynamical analysis of gene regulatory networks with delays. ComPlexUs 3: 231–251.

12. Willey JM, Sherwood L, Woolverton CJ (2011) Prescott's microbiology. McGraw-Hill.

13. Bennett MR, Pang WL, Ostroff NA, Baumgartner BL, Nayak S, et al. (2008) Metabolic gene regulation in a dynamically changing environment. Nature 454: 1119–1122.

14. Smith O (1957) Closed control of loops with dead time. Chem Eng Progress 57: 217–219.

15. Locke JC, Elowitz MB (2009) Using movies to analyse gene circuit dynamics in single cells. Nature Reviews Microbiology 7: 383–392.

16. (2011) Segmentation, tracking and lineage analysis of yeast cells in bright field microscopy images.

17. Pedersen SJK (2007) Circular hough transform. Aalborg University, Vision, Graphics, and Interactive Systems .

18. Ferry M, Razinkov I, Hasty J (2011) Microfluidics for synthetic biology from design to execution. Methods Enzymol 497: 295.

Supplementary Informations Captions

Figure S1. IRMA hybrid model. A hybrid model featuring two distinct vector fields (F_1 and F_2) has been derived from the model presented in [1]. As long as Glucose is administered ($u = 0$) F_1 is activated, while the system switches to F_2 as soon as Galactose is added to the medium to reflect the inner dynamics of the synthetic circuits to be controlled.

Figure S2. IRMA control scheme. The upper block scheme represents the control algorithm. The lower block magnifies the Predictor block referred to as $Pred$ in the previous schematic. The y_{ref} signal sets the desired output y for the controlled system P. The prediction block ($Pred$) uses the input u and output y related to the actual plant P to compute an anticipated version of the output obtained by simulating the response $\hat{y}$ of mathematical model of P in which $\tau = 0$. This signal is immediately used to assess the effectiveness of the control action by feeding it back to the first comparator that computes the error e made by the system. Moreover, the actual output y of the plant, is compared with a delayed version of the $\hat{y}$ signal (as effect of the $e^{-\tau}$ block contribution) to account for discrepancies between the predicted (via IRMA's model M) and real plant behavior. A low-pass filter meant to suppress high-frequency noise is applied to the resulting signal to obtain (y_s) that is finally fed back to the comparator that will subtract it from y_{ref} so as to obtain the control error e.

Figure S3. Cohen-Coon approximation for IRMA. In order to design a suitable PI controller we estimated three parameters, namely Θ, μ and d (as referenced in [6]) from the step response profile of the IRMA nonlinear model in equation 1-5. The solid blue line represents the response of our gene network (Cbf1p being the output) to the addition of Galactose to the growth media at $t = 0$ s while the dashed blue line shows the same information for the time delayed linear system identified with the method in [6].

Figure S4. Finite State Automaton implementing the control algorithm in Supplementary Figure S2. In the initial state, state 0, the calibration is carried out as previously described. The system cycles on this state until the initialization is completed and then moves to state 1. At this point given the error e, the PI - PWM block is simulated to compute the control input u. In state 2 the model prediction is calculated given u; the input is then applied to the physical system by means of hydrostatic pressure modulation in step 3 (the correct amounts of Galactose/Raffinose and Glucose are provided at the end of this step). In state 4 the delayed version of computed output is calculated; during state 5, the presence of a new image is verified, and the image processing algorithm is run in order to obtain the system output measure. Given this it is possible to calculate y_s and the error e for the next control iteration. The algorithm then moves to state 1 for a new control iteration to start.

Figure S5. In-silico prediction-based signal tracking control of IRMA. The predictor-based algorithm is applied to control the dynamical model of IRMA to a time varying reference signal (y_{ref}, in blue); the computed control input (higher state standing for Galactose and lower state meaning Glucose providing) is represented in red (u). The good overlap between the reference signal and the simulated Cbf1 time evolution (y) provides evidence for the robustness of the designed control scheme in two cases: (top panel) with no delay ($\tau = 0$ min) and with $\tau = 100$ min (bottom panel).

Figure S6. In-silico prediction-based set point control of IRMA. The predictor-based algorithm is applied to control the dynamical model of IRMA to a constant reference signal (y_{ref} in blue). The set point is calculated as the 80% of the maximum value for the simulated Cbf1 time evolution evaluated until $t = 0$min. The control input (computed after time 0 where higher state standing for Galactose and lower state meaning Glucose providing) is represented in red (u). The simulation was performed with the dynamical model without delay (top panel) or with a delay $\tau = 100$ min (bottom panel). In both cases, the control action is able to guarantee good dynamical performances of the system, indeed the simulated Cbf1 time evolution (y in green) tightly matches the reference signal.

Figure S7. In-silico PI/PWM signal tracking control of IRMA. The PI/PWM control algorithm is applied to control the dynamical model of IRMA to a time varying reference signal (y_{ref}, in blue); the computed control input (high level: Galactose; low level: Glucose) is shown in red (u); the Cbf1 time evolution is shown in green (y). When the control is applied to the model without the delay, the control output (y) follows the reference signal (top panel); whereas the PI - PWM is not able to achieve the control objective for the model with the delay ($\tau = 100$ min) (bottom panel).

Figure S8. In-silico PI/PWM set point control of IRMA. The PI/PWM control algorithm is applied to control the dynamical model of IRMA to a constant reference signal ($y_{ref} inblue$). The set point is equal to 80% of the maximum value for the simulated Cbf1 time evolution evaluated until $t = 0$min. The control input, computed after time 0, is shown in red (u high level: Galactose; low level: Glucose). The simulation was performed with the dynamical model without delay (top panel) or with a delay $\tau = 100$ min (bottom panel). When the control is applied to the model without delay, the control output (y) follows the reference signal (top panel); on the contrary, the PI - PWM is not able to achieve the control objective for the model with the delay ($\tau = 100$ min) (bottom panel).

Figure S9. Image processing. The algorithm applies Otsu thresholding to binarize the grey scale phase contrast image (A). Convex hulls (B) are then used to limit the application of the Circular Hought Transform to find cells' centers and edges (C).

Figure S10. In-vivo set point control experiment n. 1 for the *GAL1* promoter - fluorescence standard deviation. By using the off-line analysis described in the text, it is possible to compute the standard deviation of the fluorescence for each frame acquired during the control experiment. The desired amount of protein (y_{ref} in blue), the quantified GFP (y green line) and the standard deviation's upper and lower bounds (thin green lines) are shown; the control error (top pane in black) is computed as the difference between the feedback signal and the control reference . The input signal u computed by the control algorithm is shown in red (bottom panel).

Figure S11. In - vivo set point control experiment n. 2 for the *GAL1* promoter - fluorescence standard deviation. By using the off-line analysis described in the text, it is possible to compute the standard deviation of the fluorescence for each frame acquired during the control experiment. The desired amount of protein (y_{ref} in blue), the quantified GFP (y green line) and the standard deviation's upper and lower bounds (thin green lines) are shown; the control error (top pane in black) is computed as the difference between the feedback signal and the control reference . The input signal u computed by the control algorithm is shown in red (bottom panel).

Figure S12. In - vivo set point control experiment n. 3 for the *GAL1* promoter - fluorescence standard deviation. By using the off-line analysis described in the text, it is possible to compute the standard deviation of the fluorescence for each frame acquired during the control experiment. The desired amount of protein (y_{ref} in blue), the quantified GFP (y green line) and the standard deviation's upper and lower bounds (thin green lines) are shown; the control error (top pane in black) is computed as the difference between the feedback signal and the control reference . The input signal u computed by the control algorithm is shown in red (bottom panel).

Figure S13. In - vivo set point control experiment n. 4 for the *GAL1* promoter - fluorescence standard deviation. By using the off-line analysis described in the text, it is possible to compute the standard deviation of the fluorescence for each frame acquired during the control experiment. The desired amount of protein (y_{ref} in blue), the quantified GFP (y green line) and the standard deviation's upper and lower bounds (thin green lines) are shown; the control error (top pane in black) is computed as the difference between the feedback signal and the control reference . The input signal u computed by the control algorithm is shown in red (bottom panel).

Figure S14. In - vivo set point control experiments *GAL1* promoter - Cell count and coefficient of variation. (A-D) For each of the experiments of Supplementary Figures S10, S11, S12 and S13, the number of cells (top) and the coefficient of variation (bottom) are shown.

Figure S15. In - vivo set point control experiment for the IRMA network - fluorescence standard deviation. By using the off-line analysis described in the text it is possible to calculate the standard deviation of the fluorescence for each frame acquired during the control. The desired amount of protein (y_{ref} in blue), the quantified GFP (y green line), the standard deviation's upper and lower bounds (thin green lines) and the control error e in black are shown; mean μ, variance σ and coefficient of variation CV of the control error are also shown; the p-value was computed as described in the Supplementary Information text (top panel). The input signal u computed by the control algorithm is shown in red (bottom panel).

Figure S16. In - vivo signal tracking control experiment for the IRMA network - Cell count and coefficient of variation. For the experiment of Supplementary Figure S15, the number of cell (top panel) and the coefficient of variation (bottom panel) are shown.

Figure S17. Response to a sustained galactose input for the IRMA network. Green line: fluorescence measured when the cells are treated with galactose for the whole experiment; light green line: fluorescence measured during the in-vivo set point control experiment (Figure 7 - main text); black line: the control reference of the set-point control experiment (Figure 7 - main text); red line: the sustained galactose input provided to the cells population; light red: the input calculated automatically by the control algorithm and used to regulate the production of GFP to the desired level in in-vivo set point control experiment (Figure 7 - main text).

Figure S18. Response to a sustained galactose input for the IRMA network. Green line: fluorescence measured when the cells are treated with galactose for the whole experiment; blue line: the control reference of the set-point control experiment (Figure 7 - main text) (Top panel).(Bottom panel) red line: the sustained galactose input administered to cells. The normalised root mean square error (NRMSE) of the deviation between the blue and the green signal has been reported to be equal to 0.33

Figure S19. In - vivo signal tracking control experiment for the IRMA network - fluorescence standard deviation. By using the off-line analysis described in the text it is possible to calculate the standard deviation of the fluorescence for each frame acquired during the control. The desired amount of protein (y_{ref} in blue), the quantified GFP (y green line) and its upper and lower bound of the standard deviation (thin green lines) are plotted (top panel). The input signal u computed by the control algorithm is shown in red (bottom panel).

Figure S20. In - vivo signal tracking control experiment for the IRMA network - Cell count and coefficient of variation. For the experiment of Supplementary Figure S19, the number of cell (top panel) and the coefficient of variation (bottom panel) are plotted.

Figure S21. Internal signals of the control experiment in Fig. 8 (main text). Time evolution of the most relevant signals in the control loop are shown. In particular the Galactose concentration in the medium (u) provided to the cells has been plotted in red, while the output of the delay-free model ($\hat{y}$) and its delayed version ($\hat{y}_\tau$) are shown in green and violet respectively. The error signal e (black) calculated as the difference between y_{ref} and y_s (cyan) is also depicted; mean μ, variance σ and coefficient of variation CV of the control error are also shown.

Figure S22. In - vivo signal tracking control experiment 2 for the IRMA network - fluorescence standard deviation. By using the off-line analysis described in the text it is possible to calculate the standard deviation of the fluorescence for each frame acquired during the control. The desired amount of protein (y_{ref} in blue), the quantified GFP (y green line) and its upper and lower bound of the standard deviation (thin green lines) are plotted; the control error calculated as the difference between the feedback signal and the control reference is shown in black (top panel). The input signal u computed by the control algorithm is shown in red (bottom panel).

Figure S23. In - vivo signal tracking control experiment 2 for the IRMA network - Cell count and coefficient of variation. For the experiment of Supplementary Figure S22, the number of cell (top panel) and the coefficient of variation(bottom panel) are plotted.

Figure S24. Calibration phase. The calibration data have been reported for the experiment in Fig. 8 . The simulated (blue) and quantified (green) Gfp evolution have been used to relate fluorescence data to model predictions (model units).

Figure S25. IRMA switch off experiment. Top panel: the green signals represent the measured fluorescence during *in-vivo* switch - off experiments, the blue signal is the result of *in-silico* switch off experiment using the dynamical model of IRMA (all the experimental signals are rescaled to the model range). Bottom panel: the input used to perform the experiment; cells have been fed for 180 minutes with galactose (ON signal, 1 for the mathematical model) and for 420 minutes with glucose (OFF signal, 0 for the mathematical model).

Video S1. Movie of the experiment in Figure 8. (Top left panel) Yeast cell fluorescence during the control experiment; (top right panel) cell count; (bottom left panel) desired (y_{ref} in blue) experimentally quantified GFP fluorescence (y in green) and input (u in black) calculated by the control algorithm are shown for the whole duration of the experiment; (bottom right panel) histogram of the cell fluorescence distribution.

Video S2. Movie of the experiment in Figure 7. (Top left panel) Yeast cell fluorescence during the control experiment; (top right panel) cell count; (bottom left panel) desired (y_{ref} in blue) experimentally quantified GFP fluorescence (y in green) and input (u in black) calculated by the control algorithm are shown for the whole duration of the experiment; (bottom right panel) histogram of the cell fluorescence distribution.
